# Lancet Dynamics in Greater Horseshoe Bats, *Rhinolophus ferrumequinum*

**DOI:** 10.1371/journal.pone.0121700

**Published:** 2015-04-08

**Authors:** Weikai He, Scott C. Pedersen, Anupam K. Gupta, James A. Simmons, Rolf Müller

**Affiliations:** 1 SDU-VT International Laboratory, School of Physics, Shandong University, Jinan, Shandong, China; 2 Department of Biology-Microbiology, South Dakota State University, Brookings, SD, USA; 3 Department of Mechanical Engineering, Virginia Tech, Blacksburg, Virginia, USA; 4 Department of Neuroscience, Brown University, Providence, RI, USA; University of Salamanca- Institute for Neuroscience of Castille and Leon and Medical School, SPAIN

## Abstract

Echolocating greater horseshoe bats (*Rhinolophus ferrumequinum*) emit their biosonar pulses nasally, through nostrils surrounded by fleshy appendages (‘noseleaves’) that diffract the outgoing ultrasonic waves. Movements of one noseleaf part, the lancet, were measured in live bats using two synchronized high speed video cameras with 3D stereo reconstruction, and synchronized with pulse emissions recorded by an ultrasonic microphone. During individual broadcasts, the lancet briefly flicks forward (flexion) and is then restored to its original position. This forward motion lasts tens of milliseconds and increases the curvature of the affected noseleaf surfaces. Approximately 90% of the maximum displacements occurred within the duration of individual pulses, with 70% occurring towards the end. Similar lancet motions were not observed between individual pulses in a sequence of broadcasts. Velocities of the lancet motion were too small to induce Doppler shifts of a biologically-meaningful magnitude, but the maximum displacements were significant in comparison with the overall size of the lancet and the ultrasonic wavelengths. Three finite element models were made from micro-CT scans of the noseleaf *post mortem* to investigate the acoustic effects of lancet displacement. The broadcast beam shapes were found to be altered substantially by the observed small lancet movements. These findings demonstrate that—in addition to the previously described motions of the anterior leaf and the pinna—horseshoe bat biosonar has a third degree of freedom for fast changes that can happen on the time scale of the emitted pulses or the returning echoes and could provide a dynamic mechanism for the encoding of sensory information.

## Introduction

Bat biosonar systems are diverse across species. The biosonar of different species of bats may thus be adapted for different sensing tasks, and individual species may adopt different sensing strategies and achieve a high performance through sensory specialization. Horseshoe bats (family *Rhinolophidae*), for example, are a group of bats that emit compound constant-frequency-plus-frequency-modulated (cf/fm) echolocation sounds. These bats fly and hunt for insects in confined natural spaces, such as near vegetation, that pose substantial challenges from background clutter for sonar-based navigation and prey capture [1–3]. With these unusual abilities comes a particularly sophisticated biosonar system, that includes specializations of signal design (cf narrow band pulse segments [4])), the cochlea (acoustic fovea [5]), and for rapid, adaptive control of the received carrier frequency (Doppler-effect compensation [6]).

In addition to these specializations, horseshoe bats are notable for emitting their ultrasonic pulses through the nostrils instead of through the mouth. These nasal passages act as a band-pass filter that leaves the second harmonic (∼78 kHz in the case of the bats in the present study) to dominate the emitted sonar pulse [7]. As is the case of other nasal-emitting bats, the nostrils are surrounded by conspicuous fleshy baffles, comprising the “noseleaf”, that diffract the outgoing ultrasonic waves and shape the broadcast beam. Unlike man-made sound-emission baffles, such as the horns used for megaphones or loudspeakers, the noseleaves of horseshoe bats have fairly complex shapes that consist of three parts: (i) the anterior leaf, an incomplete conical baffle around the anterior and lateral rims of the nostrils, (ii) the sella, a peg positioned in the center of the noseleaf posterior of the nostrils and the (iii) lancet, a triangular process that makes up the top (posterior) portion of the noseleaf. The lancet’s shape has been shown to have an influence on the width of the biosonar beam, although different studies have produced different results as to whether this effect is a widening [8] or a narrowing [9].

Recent results have suggested the existence of dynamic processes in the biosonar beams of horseshoe bats and the mechanical diffraction mechanisms that produce them: On the reception side, it has been shown that greater horseshoe bats can deform the shape of their outer ears (pinnae) by bending the pinna tip over a distance of about 15 percent of the total pinna height [10]. This transition can happen within about one tenth of a second and changes the pinna’s reception beampattern qualitatively from being dominated by single main-lobe to having significant sensitivity outside the main lobe [10].

Effects of non-rigid deformation of beamforming baffle structures have also been demonstrated on the emission side of the horseshoe-bat biosonar system: Greater horseshoe bats have been recorded making twitching motions of the anterior leaf [11]. These motions altered the curvature of the anterior leaf’s surfaces and produced changes in the overall noseleaf geometry that amount to up to about one quarter of a wavelength [11]. The motions of the anterior leaf occurred on an even shorter time scale than those of the pinna and were tightly correlated with the emission of the biosonar pulses. These motions started with the onset of the emission and were completed around the end of the pulse. At present, it remains unclear if these fast changes have a functional significance and what it could be.

Changes to the beamwidth in echolocating bats have been observed in Vespertilionid bats, which emit FM sonar sounds through the open mouth [12], as well as in Japanese greater horseshoe bats (*Rhinolophus ferrumequinum nippon*) [13]. In both cases, increases in beamwidth were seen as the bats approached their prey. This has been interpreted as a strategy to counteract evasive maneuvers of the prey by widening the field of view to avoid losing the target. For the FM-emitting Vespertilionid bats, the increase in beamwidth can be explained by a lowering of the pulse frequencies, which reduces the effective size of the emitting aperture [12]. However, for the Japanese greater horseshoe bats, the widening of the beam happens without a shift in frequency, and hence requires another mechanism. Since beamwidth is determined by the relationship between wavelength and aperture size, it is hypothesized that the beamwidth change in the horseshoe bats is the result of a change in the geometry of the noseleaf. Furthermore, since the observed beam widening took the form of a continuous change across many sequential pulses [13], the implied changes in noseleaf shape should also take place across many pulses i.e., be slow compared to the pulse emission intervals. The fast, periodic changes that have been observed in the anterior leaf of horseshoe bats [11] do not fit this multiple-pulse pattern because they are completed within a single pulse. However, it is still possible that horseshoe bats vary properties of the twitching motion (e.g., the displacement amplitude) continuously on a slower time scale over a sequence of pulses.

In horseshoe bats, it has been observed by the authors and others that while emitting biosonar pulses at least one other portion of the noseleaf is conspicuously in motion. This part is called the lancet and it makes up about 30 percent of the noseleaf’s surface area. Little experimental data exists on the acoustic contributions of the lancet in *Rhinolophus ferrumequinum* so far, and only anecdotal observations can be found in the literature [14].

Therefore, the goals of the research reported here were to: i) Establish whether there is relationship between lancet motion and ultrasonic pulse emission. If lancet motion were uncorrelated with pulse emissions or if it occurred prominently in the gaps between successive biosonar pulses, it would be difficult to argue that this motion could have functional relevance for biosonar. ii) Investigate the effects that movements of the lancet may have on the emitted pulses in this dynamic biosonar system. Because direct acoustic measurement of potentially subtle changes in broadcast beamwidth would be disrupted by large movements of the bat’s head during recording, 3D digital models of the noseleaf were developed and subjected to movements of the lancet while finite-element computations were used to estimate the emission beampatterns.

## Results

**Fig 1:**
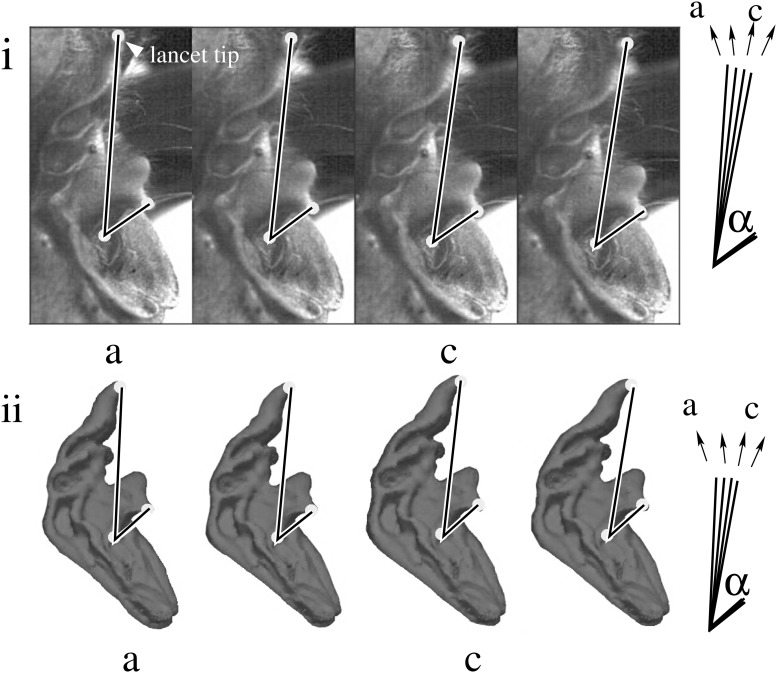
Deflection pattern of the lancet. Four sequential frames (*a*, *b*, *c*, *d*) from a high-speed video recording of lancet movement (upper panel i) and the corresponding digital shape models (*a*′, *b*′, *c*′, *d*′; lower panel ii). These data (from male 1) depict a representative example of the lancet motion that was observed in all individual bats studied here. In each case, *a*, *a*′ are in the resting (upright) position, *b*, *b*′ are flexed forward 4°, *c*, *c*′ are flexed forward 8°, and *d*, *d*′ are maximally flexed at 12°.

**Fig 2:**
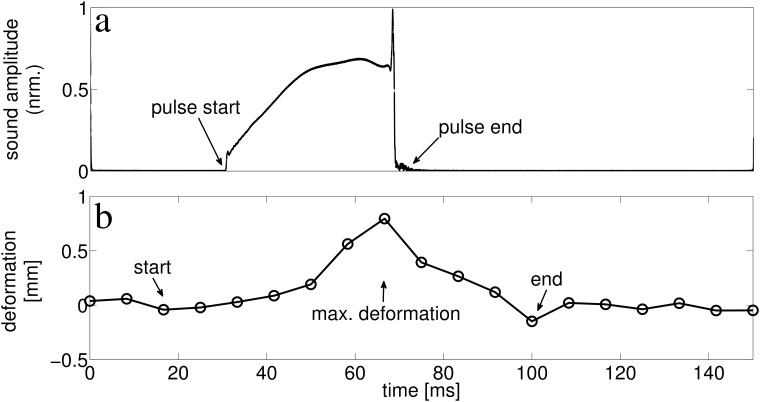
Example of the temporal relationship between lancet movement and the respective biosonar pulse. a) envelope of the sound pressure amplitude, b) lancet displacement amplitude. The abscissa (time) is the same in both a and b.

Quantitative measurement of the lancet’s motion was implemented with synchronized recording using two high speed video cameras and an ultrasonic recording system with its microphone at a distance of 15 cm from the bat’s nostrils. Three landmarks were selected on the noseleaf (lancet tip, sella tip, and sella base, see Fig. 1i) and used to obtain the relative motion of lancet by stereo triangulation. By tracing the motion of lancet over time, the relationship between lancet motion and sonar pulse occurrence was determined for 27 out of 46 recorded biosonar sequences (13 sequences had no conspicuous lancet deformation, 6 showed some motions but the recordings had insufficient quality for three-dimensional tracking) which were subjected to statistical analysis (see Fig. 2 for an example, Table 1 for a summary of the statistics).

**Table 1:** Statistical characterization of the lancet motions observed in the analyzed recordings.

N = 27	pulse duration [ms]	lancet motion duration [ms]	max. tip displacement [mm]	max. lancet rotation angle [°]
average	33.67	67.47	0.91	9.22
standard deviation	7.14	11.99	0.21	2.30

Frame-by-frame comparisons of the video recordings revealed that the lancet carries out a motion that is directed forward, away from the head, while the nostrils stayed approximately in place. The lancet rotated forward, then backward, returning approximately to the original position relative to the bottom edge of the noseleaf’s sella that was as a fixed reference to describe the lancet’s rotation relative to the overall position and orientation of the noseleaf (see Fig. 1i). The angular rotation of the lancet was found to be 9.23° on average with a maximum observed value of about 12° and a standard deviation of 2.30°. This rotation is equivalent to an average linear displacement of the lancet tip of 0.91 mm (maximum: 1.17 mm, standard deviation: 0.21 mm, see Table 1). The lancet motion thus resulted in a non-rigid deformation of the overall noseleaf shape that increased the curvature of the noseleaf’s aggregate forward-pointing surface. These lancet rotations were observed to be coupled with pulse emission but occurred on a somewhat longer time scale than the duration of individual sounds (see Fig. 2). The duration of the lancet motion had a mean of 67.47 ms (standard deviation 11.99 ms), while the duration of the pulses had a mean of 33.67 ms (standard deviation 7.14 ms, Table 1). The mean of the flexion velocity was 3 cm/s with a standard deviation of 1.6 cm/s.

**Fig 3:**
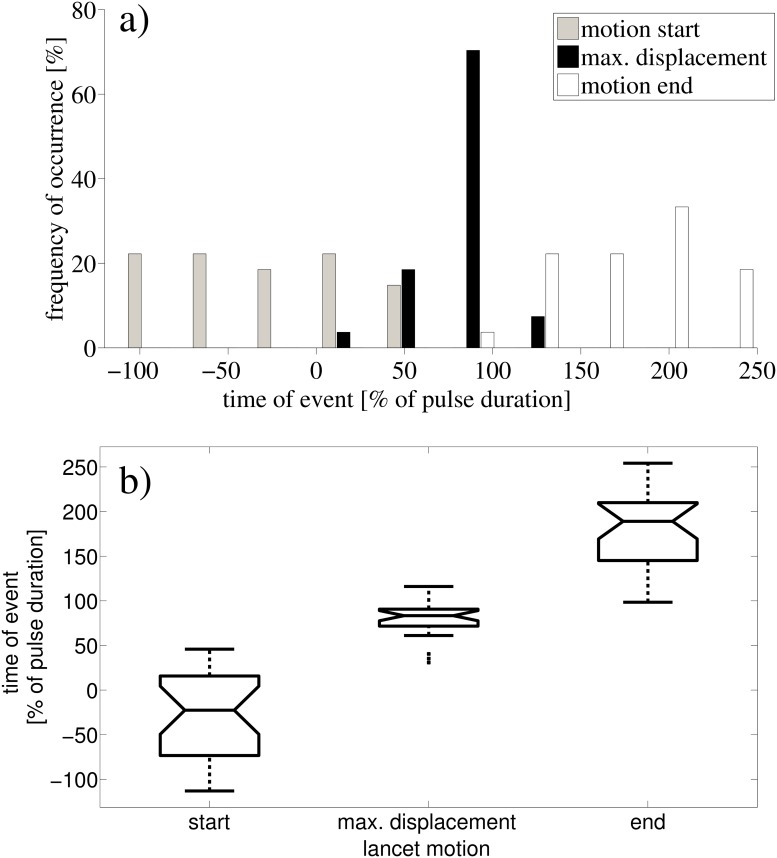
Timing statistics for the occurrence of the lancet deformation relative to the biosonar pulses. a) distributions (histograms) of the times for lancet motion start, maximum tip deflection, and motion end. b) box-and-whisker plots of the same data (*N* = 27), the center of each box shows the respective mean, box outlines the standard deviation (sd), and the extended lines show the entire data range. The mean start time is -28% (sd = 50%); mean time of the max. deflection is 79% (sd = 21%); mean end time is 179% (sd = 44%) of the pulse duration. These distributions are statistically different enough (1-way ANOVA, df = 2, F = 179, *p* < 0.01) to reject the null hypothesis that there is no synchronization. In particular, the occurrence time of the maximum deflection is tightly confined to the time interval that precedes the end of the pulse.

Across all analyzed lancet motions and pulses (*N* = 27), the lancet motions always overlapped with the emission of the pulses in time. Motions of the lancet relative to the other parts of the noseleaf were never observed between biosonar pulses. The occurrence of the lancet’s maximum flexion fell within the duration of an emitted biosonar pulse in about 90% of the pulses in the analyzed data set. In about 70% of the cases, the occurrence of the lancet’s maximum flexion fell within the terminal third of the pulse duration (see Fig. 3). As a result, the surface of the lancet was actively moving in the distal or forward direction for most of the duration of a typical pulse. The location of the maximum lancet displacement relative to the pulse duration had a much lower variability (standard deviation 21%) than for the start and end of the motion (standard deviations 50% and 179%). About 60% of the time, the lancet motion began earlier than the pulse start time, while about 95% of the time, the lancet’s deformation ended later than the end of the pulse. However, most of the sound pulse duration was covered within the duration of the lancet’s motion. Not all pulses in the biosonar sequences recorded during the experiments were accompanied by discernible lancet motion. For many biosonar pulses, the shape of the noseleaf did not change. However, whenever changes in the lancet shape occurred, these changes overlapped with the biosonar pulses in time. Hence, these results demonstrated close temporal correlations between the emitted pulses and the motion of lancet.

**Fig 4:**
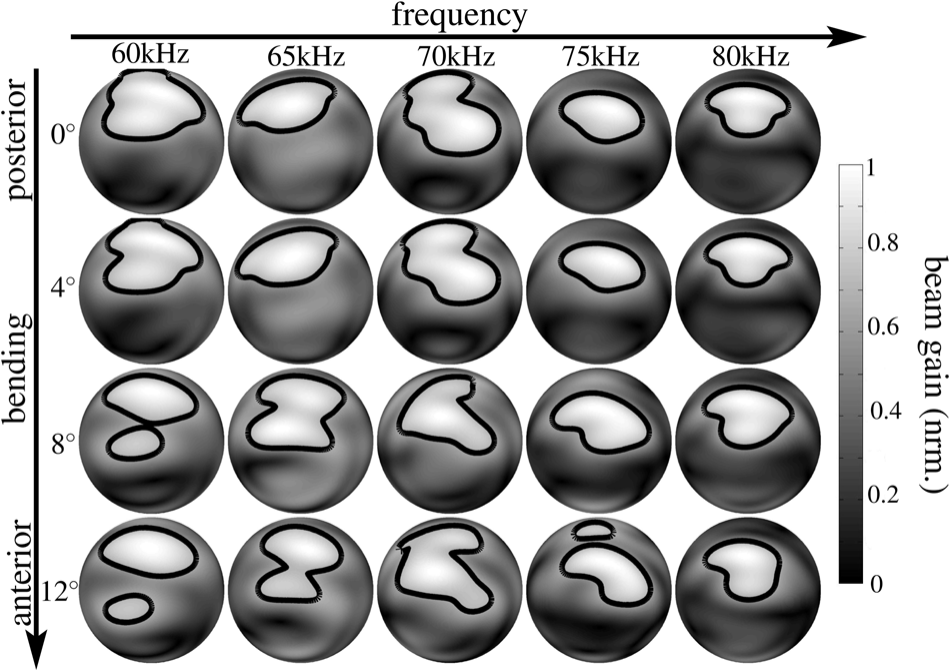
Numerical beampattern estimates obtained for the lancet rotation in the noseleaf model derived from specimen male 1. Each row represents a different orientation of the lancet from complete extension (posterior, 0°, top row) to the maximum flexion studied (anterior, 12°, bottom row). The columns correspond to different frequencies (60 kHz to 80 kHz). The level for the single contour line shown is -3 dB. The gray-level coding of the amplitude values is linear.

**Fig 5:**
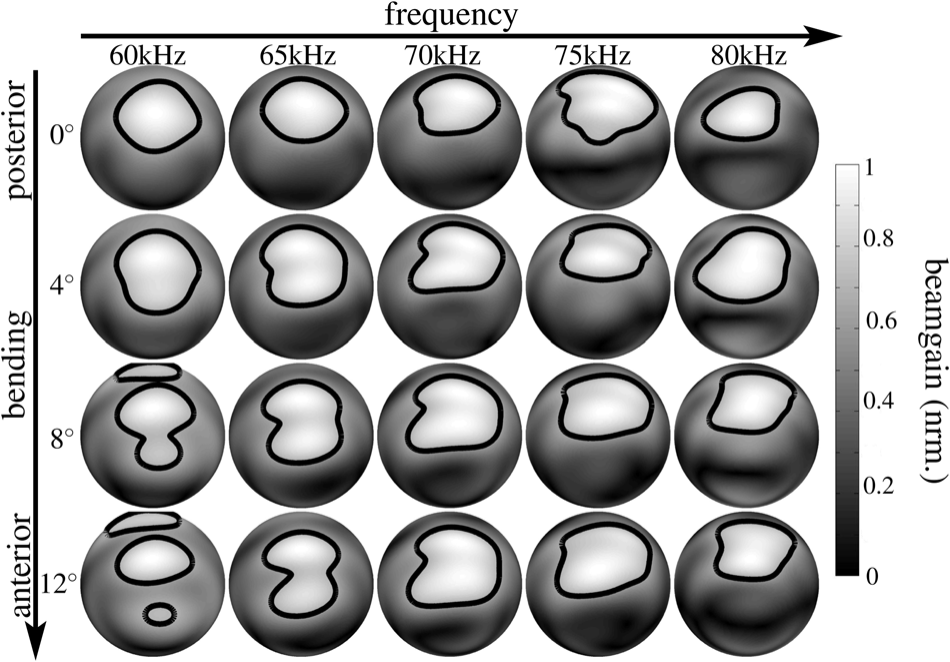
Numerical beampattern estimates obtained for the lancet rotation in the noseleaf model derived from specimen male 2. Each row represents a different orientation of the lancet from complete extension (posterior, 0°, top row) to the maximum flexion studied (anterior, 12°, bottom row). The columns correspond to different frequencies (60 kHz to 80 kHz). The level for the single contour line shown is -3 dB. The gray-level coding of the amplitude values is linear.

**Fig 6:**
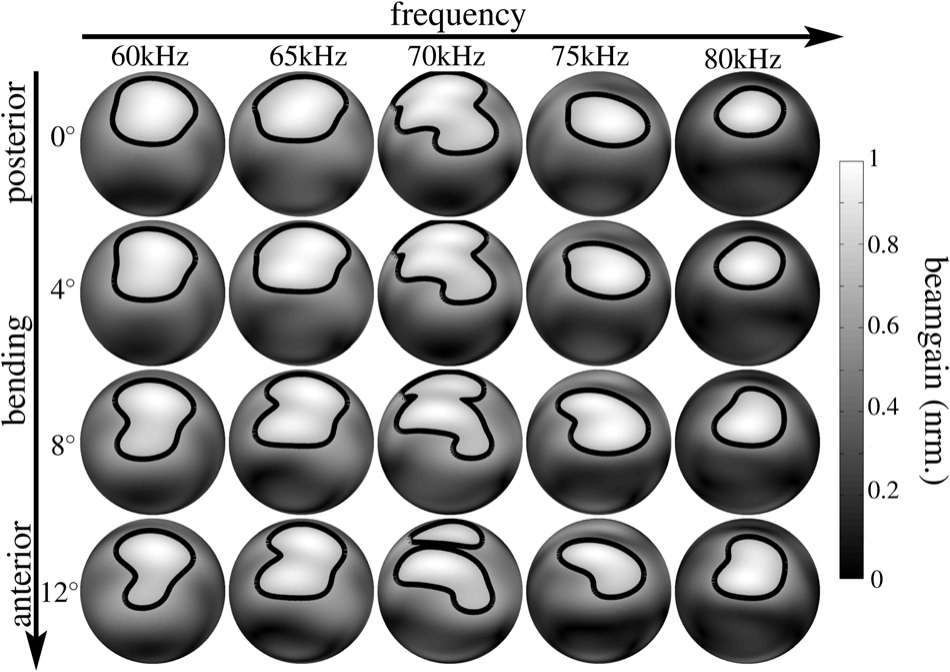
Numerical beampattern estimates obtained for the lancet rotation in the noseleaf model derived from specimen female 1. Each row represents a different orientation of the lancet from complete extension (posterior, 0°, top row) to the maximum flexion studied (anterior, 12°, bottom row). The columns correspond to different frequencies (60 kHz to 80 kHz). The level for the single contour line shown is -3 dB. The gray-level coding of the amplitude values is linear.

**Fig 7:**
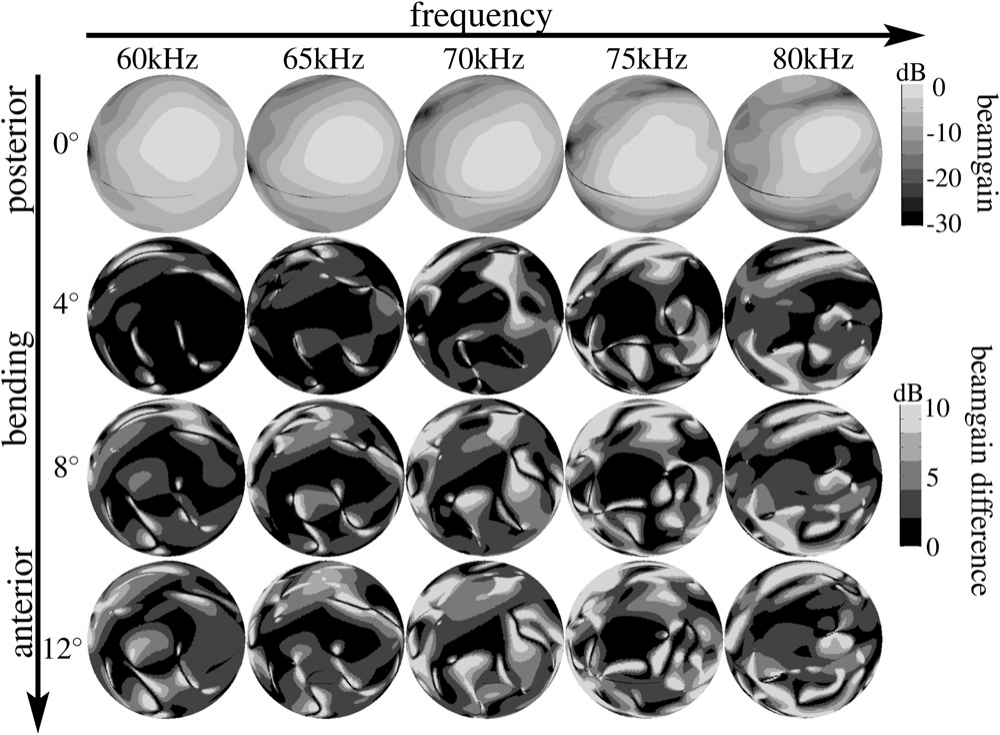
Beampattern differences induced by lancet motion for the model derived from specimen male 2. Top row: beampattern associated with the upright lancet orientation (0°). Bottom rows: differences between the beampatterns associated with the upright lancet orientation and the beampatterns associated with three successive rotations (4°, 8°, and 12°). The gray-level codings of the amplitudes and amplitude differences are logarithmic (dB-scale).

We hypothesized that the bending of the lancet might be used by horseshoe bats to actively control their spatial emission beampatterns in order to reallocate ultrasonic energy over space and time, possibly to enhance the encoding of sensory information that is of particular interest to the animals. To test this hypothesis, we employed three-dimensional digital surface mesh models of horseshoe bat noseleaf shapes derived from micro-CT scans of three individual bats (two males and one female) and rotated the lancet of the models from an initial upright position to a rotation of 12° in steps of 4° (see Fig. 1ii). For each individual 3D model, with its particular rotated version of the lancet, the resulting emission beampatterns were determined using finite element methods. Frequencies of 60, 65, 70, 75, and 80 kHz were tested to provide a representative sample of the frequency range covered by the bat’s broadcasts (60–78 kHz). The numerical beampattern predictions (see Figs. 4, 5, 6) showed profound changes in the beampattern in response to the rotations of the lancet. In general, forward rotation of the lancet resulted in emission beams that extended over a larger angle in elevation, either through a wider mainlobe or through the addition of sidelobes as is evident throughout the numerical beampattern estimates obtained for all three noseleaf models studied (see Figs. 4, 5, 6). However, besides these large-scale changes in beampattern geometry, lancet rotation also led to beampattern changes that occurred on a smaller scale, but had nevertheless a large impact on the local beampattern gain values. The prevalence of such local effects with large impacts on the beamgain is evident from the differences between the beampatterns obtained for different lancet rotations (see Fig. 7). Subtracting beampatterns obtained for the different lancet rotations studied from the beampatterns obtained for the upright lancet showed numerous local changes (see Fig. 7) with changes in beampattern gain around 10 dB not being uncommon. The prevalence of such large gain changes increased with increasing rotation of the lancet away from the upright position.

The changes observed in the numerical beampattern estimates did not show any systematic dependence on frequency other than following a general trend of beam narrowing with increasing frequency that was evident in the beampatterns obtained for all three noseleaf models (see Figs. 4, 5, 6).

## Discussion

Our data has demonstrated nonrandom movements of the lancet that are correlated with the terminal portion of the animal’s biosonar pulses. Furthermore, our numerical beampattern estimates have predicted large acoustic effects of these movements affect the emission beampattern. We had hypothesized that this flexion of the lancet has an impact on the acoustic characteristics of the noseleaf and through this characteristics could also affect the encoding of sensory information.

The lancet surface velocities estimated from the video recordings were found to be negligible compared to the propagation speed of ultrasound in air (about 343 m/s). Hence, the Doppler shifts that could be introduced into the broadcast ultrasound by the lancet motion are probably well below the frequency resolution of the horseshoe bats as manifested in auditory filter sharpness of tuning or the precision of Doppler shift compensation that was determined behaviorally [15]. Thus, Doppler shifts caused by the velocity of the lancet motion can most likely be ignored when considering a potential function of the lancet motion.

However, in contrast to the velocity, the displacement of the lancet (mean 0.91 mm, see Table 1) was found to be substantial compared to the involved wavelengths. At 78 kHz, the average frequency of the second (strongest) harmonic of the emitted calls, the wavelength in air is about 4.4 mm. Hence, the average displacement of the lancet tip corresponds to more than one fifth of the wavelength, which is readily reconciled with the substantial nature of the changes in the beampatterns that were predicted numerically.

The tight synchronization of the maximum lancet displacement with the termination of the pulse can be seen as circumstantial evidence for a possible functional role of the lancet motion. However, it is hard to interpret this finding in terms of a specific mechanism. One hypothesis could be that it is important for the lancet to be in its maximally displaced configuration for the frequency-modulated tail of the pulse. Another hypothesis could be that the continuous lancet motion throughout the constant-frequency portion of the pulse is functionally important. Both hypotheses are not mutually exclusive and the results presented here do not support of disprove either of them. It is noteworthy in this context that the changes in the beampatterns that were observed in response to the lancet rotations did not show any special patterns for frequencies that were associated with the constant-frequency component and hence the Doppler-shift compensation behavior of these animals.

With their comparatively short duration (∼67 ms on average), the observed motions of the lancet fall in a category of fast changes to the biosonar system of bats that also includes inward motions of anterior leaf [11] and non-rigid deformations of the pinna [10] in horseshoe bats. Previously reported rotations of the noseleaves in phyllostomids [16] occur on a substantially longer time scale, i.e., across multiple pulses, and have been interpreted as a steering of the beam direction through an rotation of the overall noseleaf orientation [17]. Likewise, the beamwidth changes that have been reported for horseshoe bats [13] happened over a large portion of the prey-capture sequence.

Because the motions of the lancet, anterior leaf, and pinna in horseshoe bats occur on time scales that are close to the duration of individual pulses or echoes and repeat in a cyclical manner from pulse to pulse, their potential function cannot be gradual adaptive adjustments, e.g., to beam direction or beamwidth during the approach to a prey. Nevertheless, their effect on the spatial acoustic characteristics (beampattern) of the biosonar system was found to be substantial. Hence, it could be hypothesized that the potential function of these dynamic effects has to lie in enhancing the ability of individual pulse-echo pairs to encode useful sensory information. The emission beam is well suited for this purpose as it projects a weighted illumination pattern on the bat’s environment and hence determines which scatterer contributes how much to the echo. However, the present results do not allow a determination of what would be useful sensory information to the horseshoe bats. The observed irregular patterns of large changes to the beamgains (see Fig. 7) suggest that this potential function is likely not realized through a simple reorientation or change in beamwidth, but rather depends on more intricate patterns of change in the spatial distribution of pulse energy over frequency and direction.

Since the lancet rotations were not found in about one third of the analyzed recordings, any potential function of these rotations should only pertain to certain biosonar tasks. From qualitative observations made in the course of the reported work, it appeared as if lancet rotations were elicited by novel situations, e.g., if a novel target is being presented to the bat. This impression matches what has been previously reported for ear motions in horseshoe bats [18].

The experiments reported here were designed to elicit and characterize the lancet rotations, but do not provide any information on their natural behavioral contexts, relationship to specific sonar tasks, or potential function. Understanding all these critical aspects of the lancet rotations will be reserved for future work.

## Materials and Methods

Experiments were carried out under institutional permit number 2012-377 from the Shandong University—Virginia Tech International Laboratory Animal Care Committee that specifically approved this study. Under this permit, three adult greater horseshoe bats (*Rhinolophus ferrumequinum*), two males and one female, were taken from their roosts in caves near Jinan, Shandong Province, China (geographic coordinates: 36° 40’ N, 116° 59’ E). Greater horseshoe bats have been assessed as a species of “least concern” by the IUCN Red List of Threatened Species [19]. Permission for these collections was granted by the managers of the land on which the caves were located. The bats were housed in the laboratory during the experiments and were maintained on mealworms and water (*ad libitum*). The animals were not deprived of food to influence their behavior in the experiments. During recording, the body and wings of each bat were loosely constrained by a towel so as to prevent the bats from injuring themselves and to limit any interference between respiration and sonar pulse emission. During each experiment, the bats were held on an adjustable stage that positioned the noseleaf approximately 15 cm from the recording instruments. The bats were enticed to emit sequences of biosonar pulses by presenting them with a variety of new, moving targets. The targets were chosen from everyday objects such as small balls, rulers, latex gloves, or paper cuts resembling a moth. The animals were not trained to perform any specific sonar task for the current study and no attempt was made to correlate their biosonar behavior to specific sensing task or target types.

Lancet movements were documented using two synchronized high-speed video cameras (GigaView; Southern Vision Systems, Madison, Alabama, USA) through Rodagon 50 mm-lenses (Rodenstock, Feldkirchen, Germany), using a 25 mm modular focus block (Navitar, Rochester, New York, USA) at a frame rate of 200 Hz. Three prominent landmarks (lancet tip, sella tip, sella base, see Fig. 1i) on the noseleaf were used for tracing the motion of lancet. A stereo calibration method based on checkerboard patterns [20] was used to compute the system parameters (intrinsic and extrinsic parameters of each camera, rotation and translation matrix between two cameras). The motion of landmarks on the noseleaf was obtained by the method of triangulation using the stereo view provided by the two cameras, analogous to binocular vision as has been done for the bat pinna previously [10]. Bat-produced sounds were recorded with a MEMS microphone (Momimic; Dodotronic, Castel Gandolfo, Italy). The analog output signals of the two high-speed video cameras and the sound measurement amplifier were digitized via three synchronized input channels of a data acquisition system (RIO platform, NI R7852; National Instruments, Austin, Texas, USA) with the sampling rate set to 500 kHz for each channel.

A 3D digital surface mesh model representation of the shape of noseleaf samples from three horseshoe bats was obtained using a micro-CT machine (Skyscan 1072; Bruker micro-CT, Kontich, Belgium). The individual 3D digital models were deformed (using the software 3DS Max; Autodesk, Mill Valley, CA, USA) to provide a good fit to the subsequent frame positions of the bend lancet from video recordings of lancet rotation (see Fig. 1). The 3D mesh models of noseleaf were used to predict the acoustic near-field numerically using a finite element method [21] at a resolution of 0.107 mm. At this resolution, the structure was sampled with about 40 elements per wavelength at the highest frequency simulated (80 kHz, i.e., 4.3 mm wavelength). The far-field beampatterns were obtained from the near-field results using a projection method based on the Kirchhoff integral [21].

## Ethics Statement

All animal work has been conducted in accordance with the relevant guidelines and regulations of the People’s Republic of China. Permission for animal collections was granted by the managers of the land on which the caves were located and the animals were taken from their roosts. Greater horseshoe bats have been assessed as a species of “least concern” by the IUCN Red List of Threatened Species [19]. Experiments were carried out under institutional permit number 2012-377 from the Shandong University—Virginia Tech International Laboratory Animal Care Committee that specifically approved this study.

## Supporting Information

S1 Fig Complete set of high-speed video sequences analyzed with sound recordings and motion analysis. For each of the 27 sequences analyzed, a sequence of frames from the high-speed video recording that covers observed the lancet motion is shown. Along with each video sequence, graphs of three time signals are provided: the envelope of the ultrasonic pulses recorded in parallel with the videos, the change in lancet angle, and the displacement of the lancet tip. In the graph of the lancet rotation angle, the start, maximum displacement, and end of the lancet motion are indicated with letters “s”, “m”, and “e” respectively.
(PDF)
