# Supplementary material for: Lancet Dynamics in Greater Horseshoe Bats, Rhinolophus ferrumequinum
Source: PLoS One. 2015 Apr 8;10(4):e0121700. doi: 10.1371/journal.pone.0121700 (PMC4390203; doi:10.1371/journal.pone.0121700)
Supplement: S1 Fig — For each of the 27 sequences analyzed, a sequence of frames from the high-speed video recording that covers observed the lancet motion is shown. Along with each video sequence, graphs of three time signals are provided: the envelope of the ultrasonic pulses recorded in parallel with the videos, the change in lancet angle, and the displacement of the lancet tip. In the graph of the lancet rotation angle, the start, maximum displacement, and end of the lancet motion are indicated with letters “s”, “m”, and “e” respectively. (PDF) [file pone.0121700.s001.pdf]

High-Speed Video, Sound Envelopes, & Lancet Rotations
Original Data & Primary Lancet Motion Analysis

W. He, S.C. Pedersen, A.K. Gupta, J.A. Simmons, R. Müller

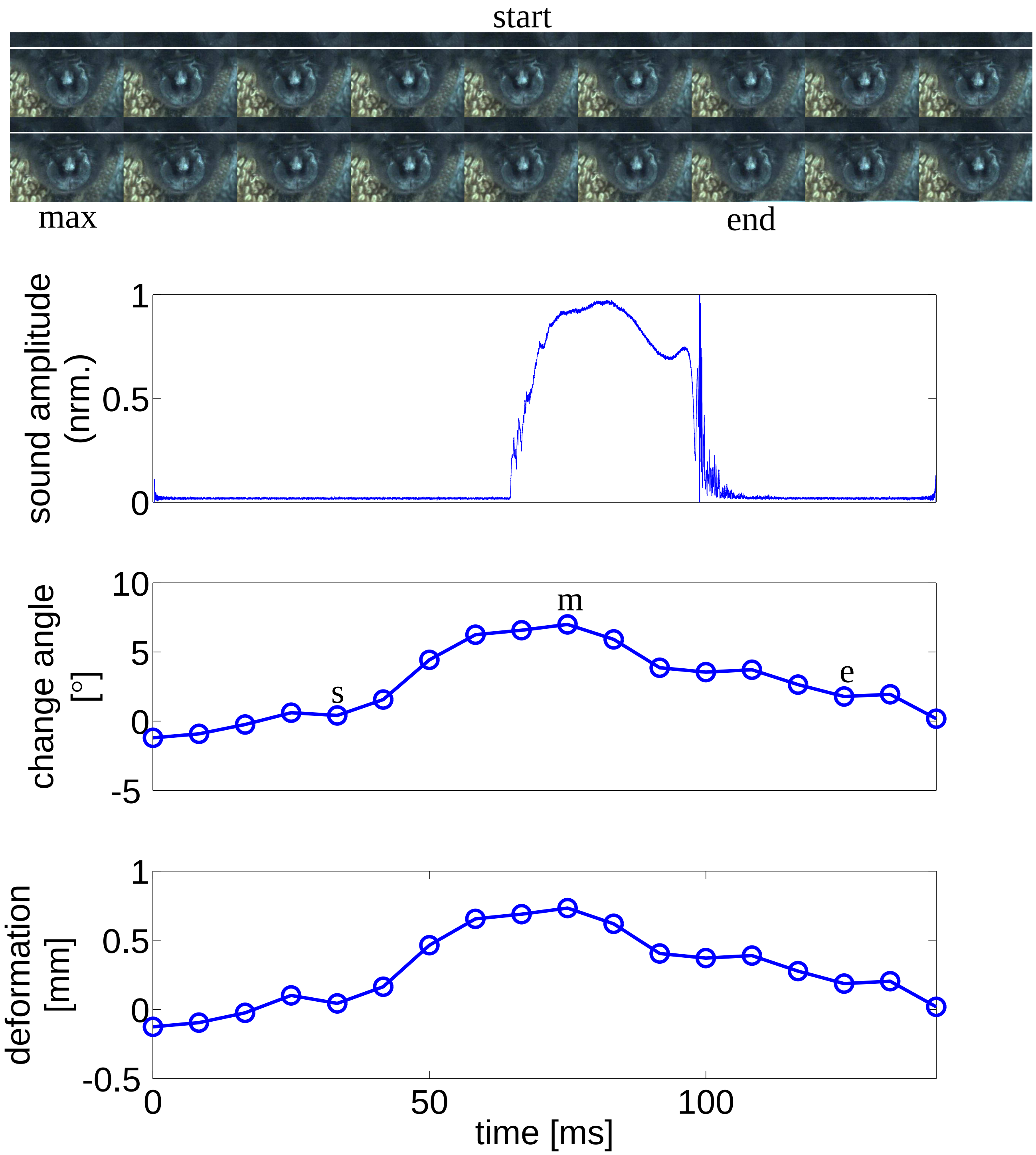

Recorded lancet motion sequence number 1.

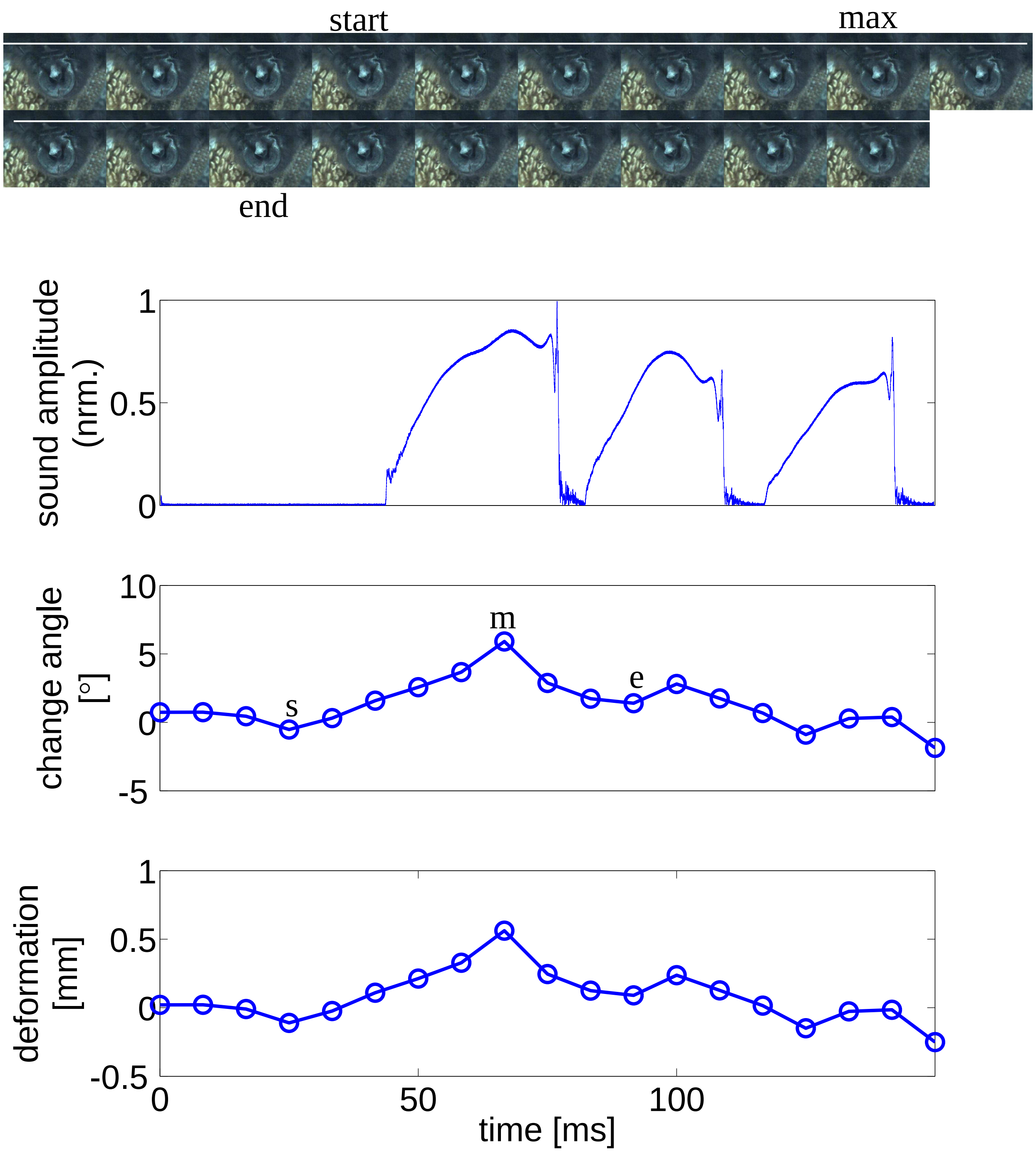

Recorded lancet motion sequence number 2.

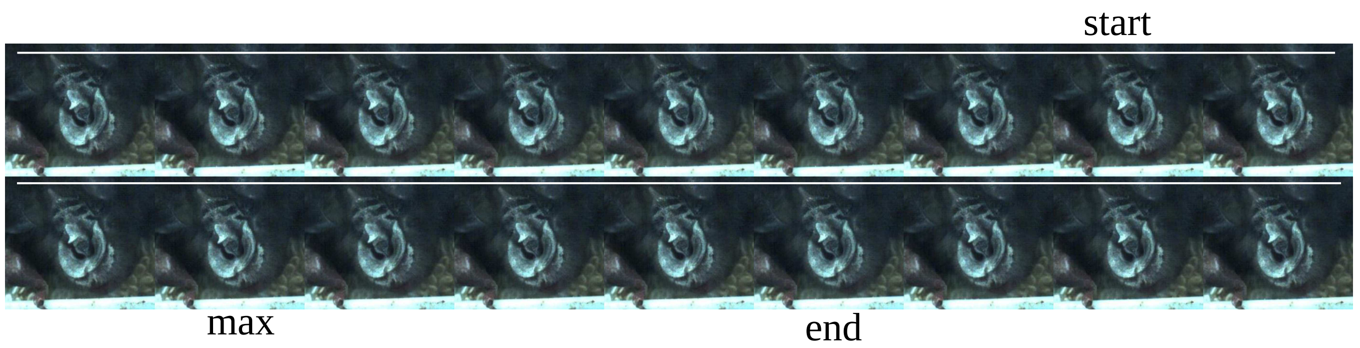

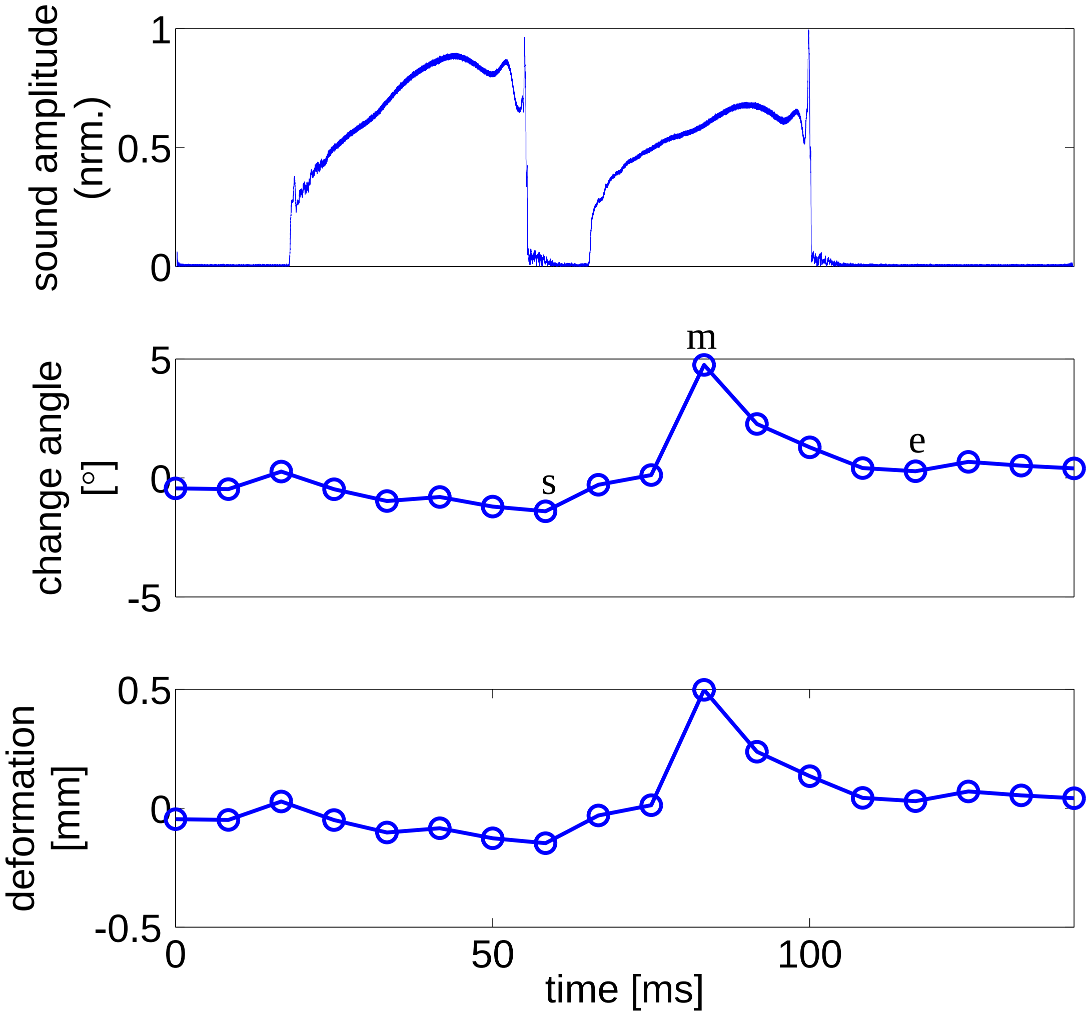

Recorded lancet motion sequence number 3.

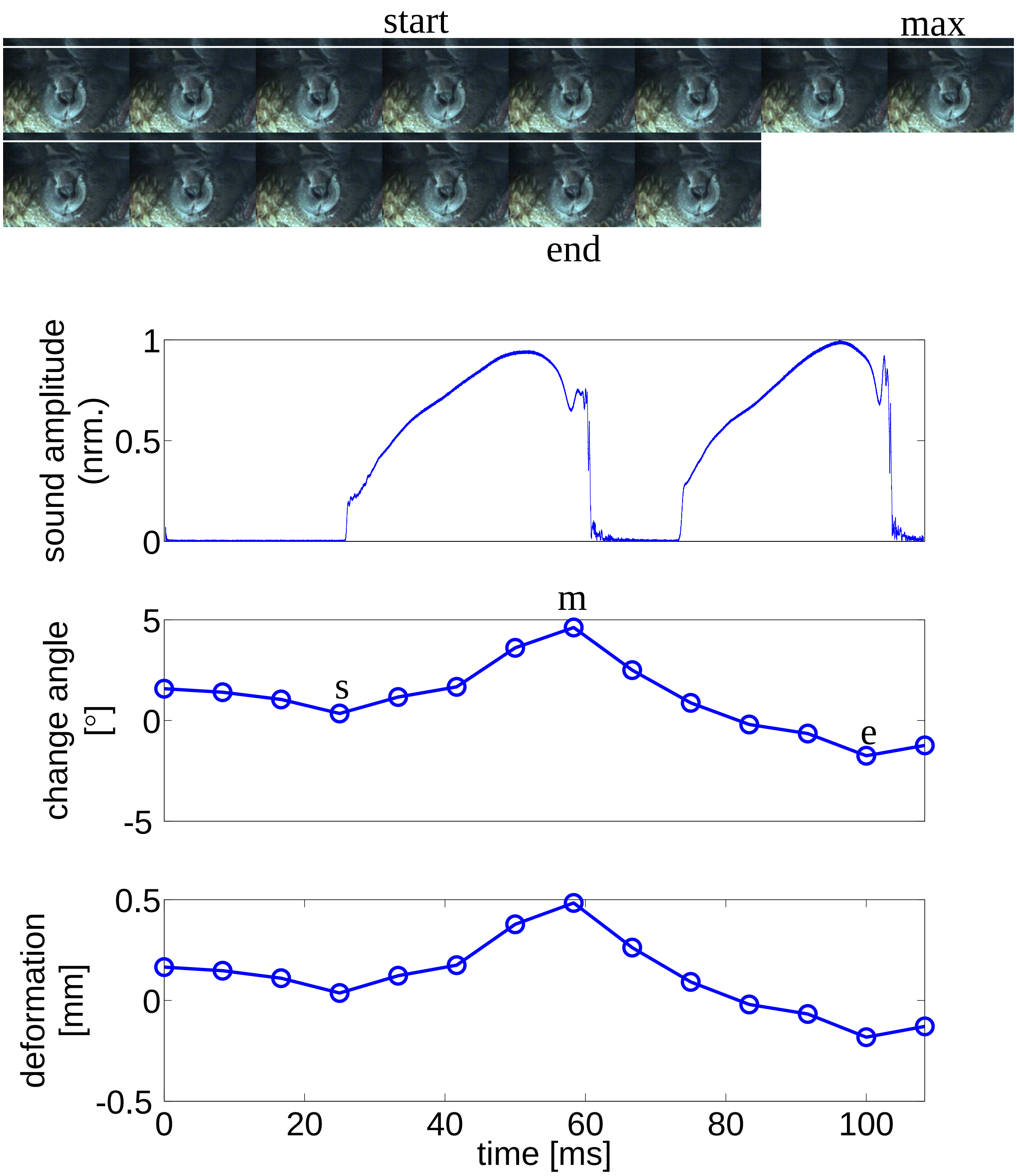

Recorded lancet motion sequence number 4.

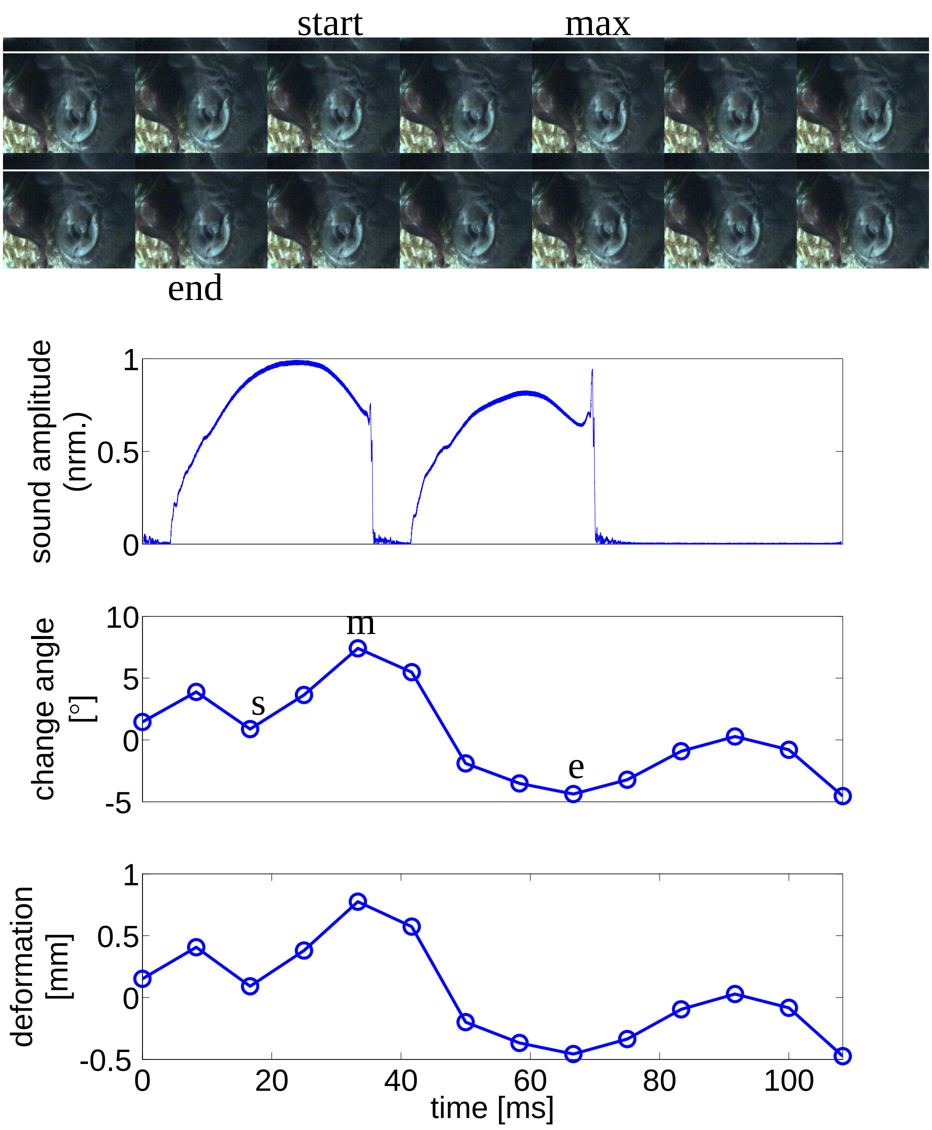

Recorded lancet motion sequence number 5.

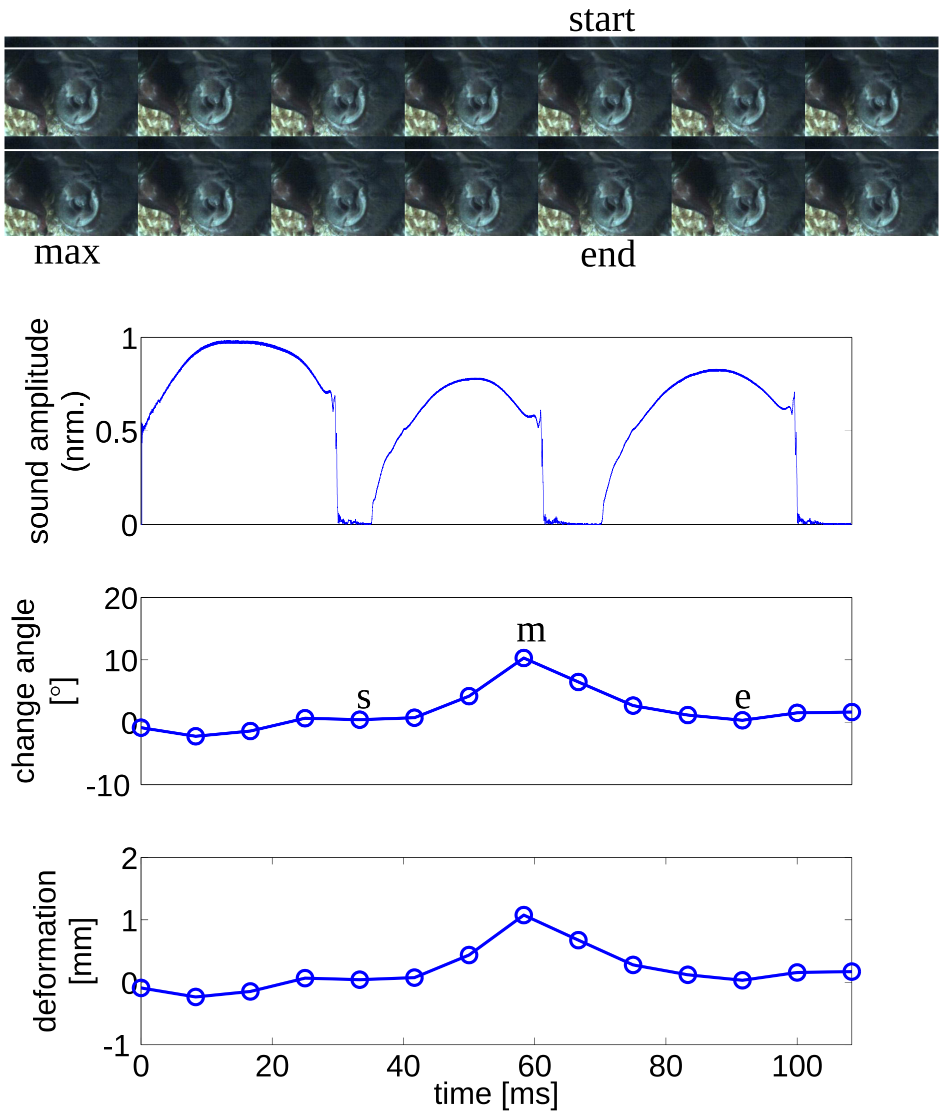

Recorded lancet motion sequence number 6.

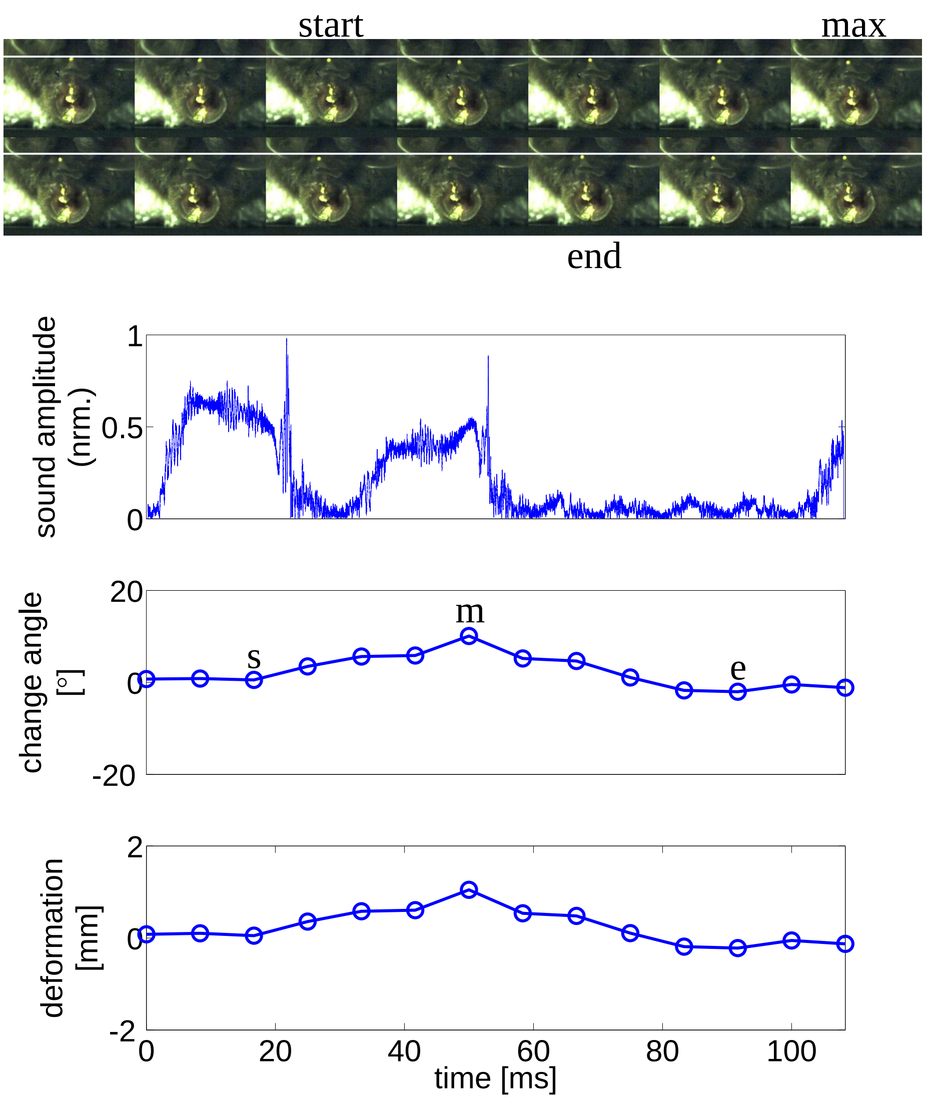

Recorded lancet motion sequence number 7.

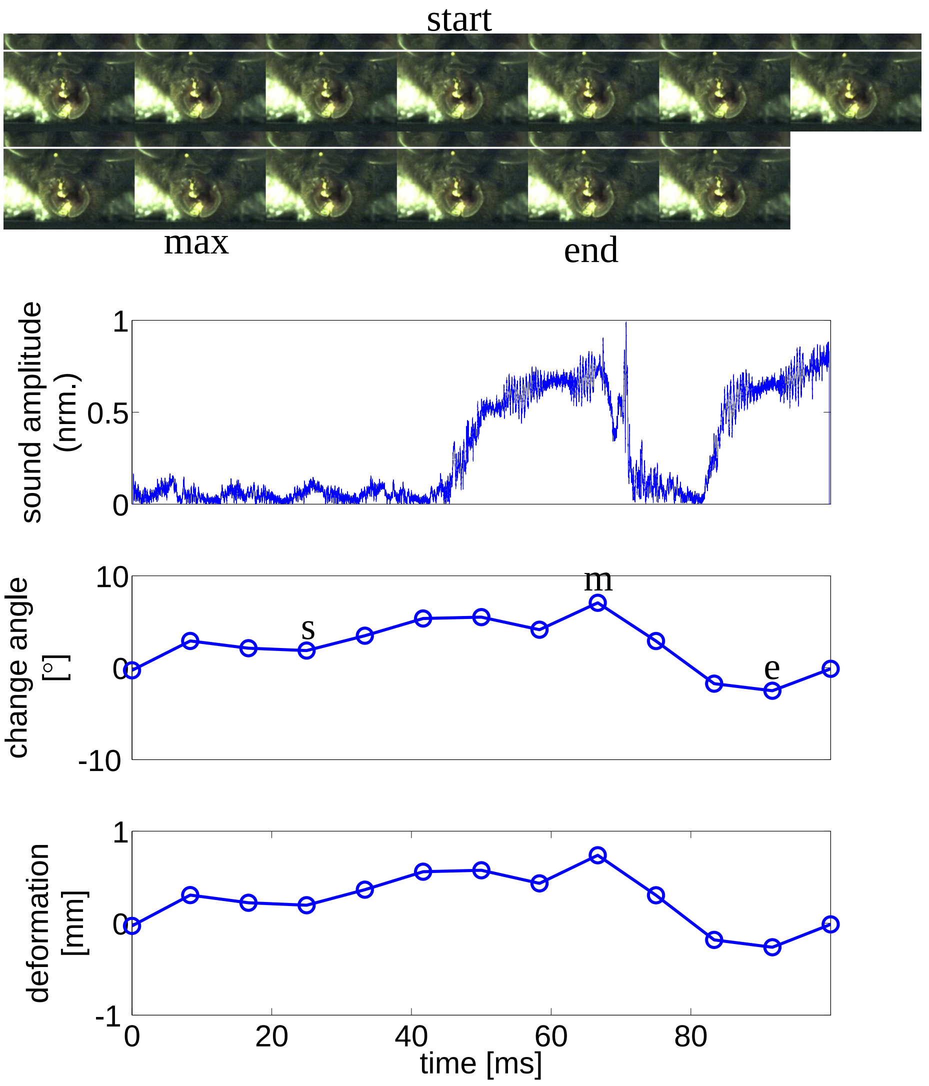

Recorded lancet motion sequence number 8.

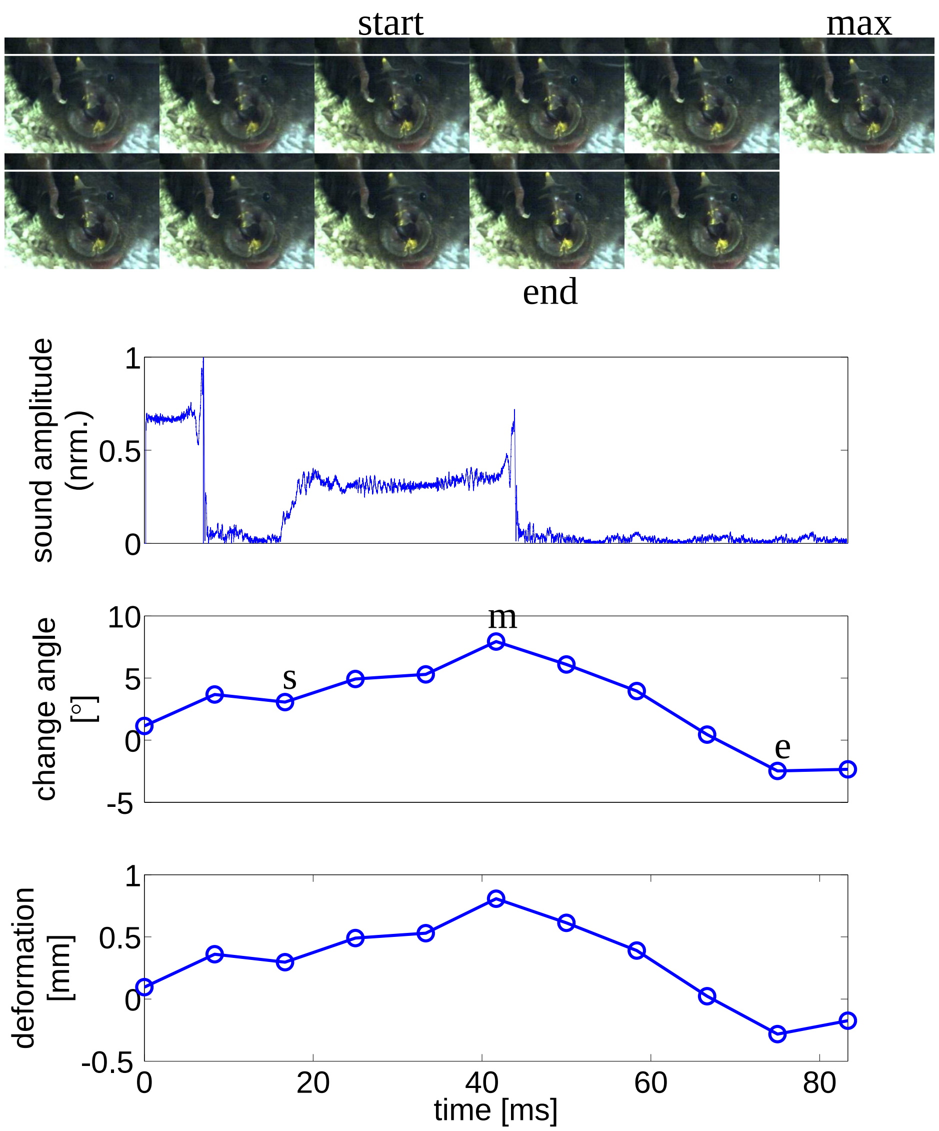

Recorded lancet motion sequence number 9.

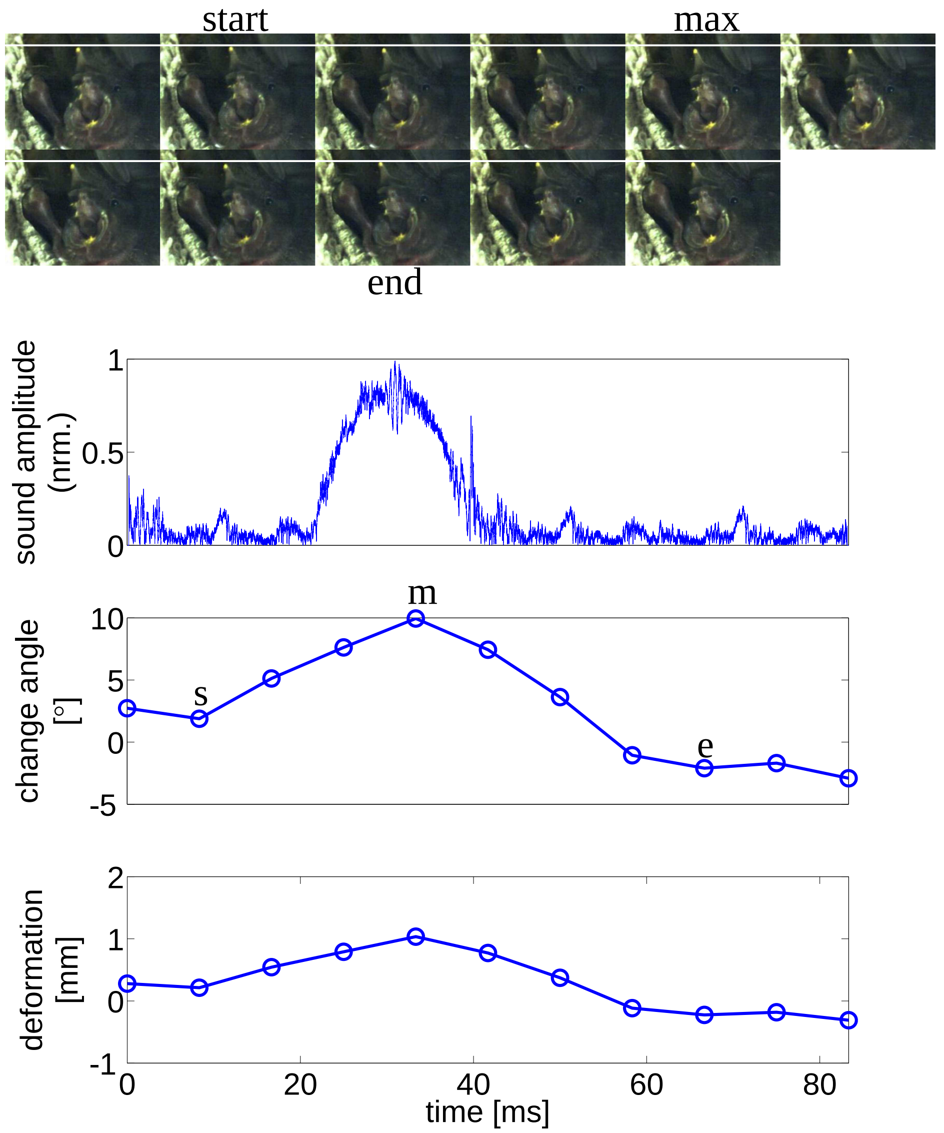

Recorded lancet motion sequence number 10.

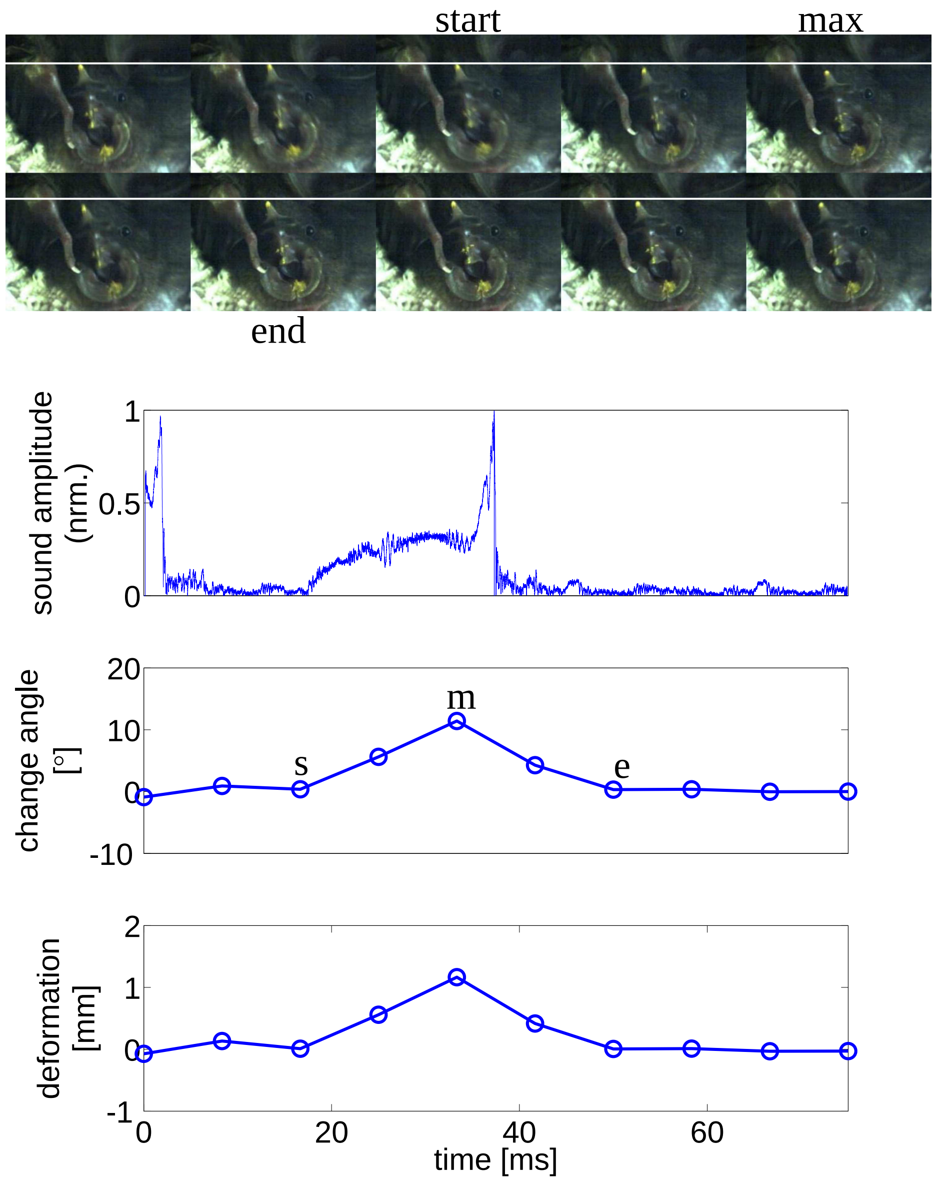

Recorded lancet motion sequence number 11.

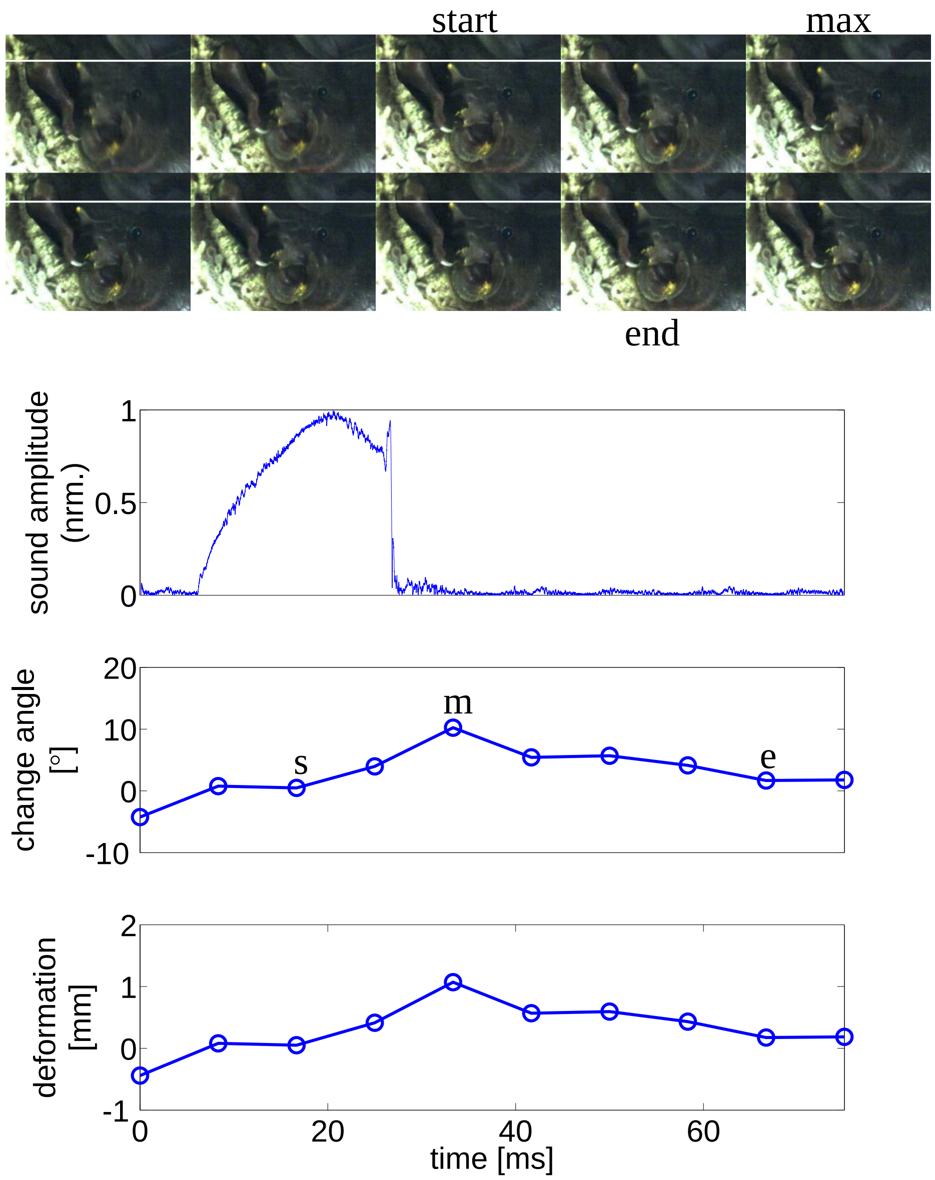

Recorded lancet motion sequence number 12.

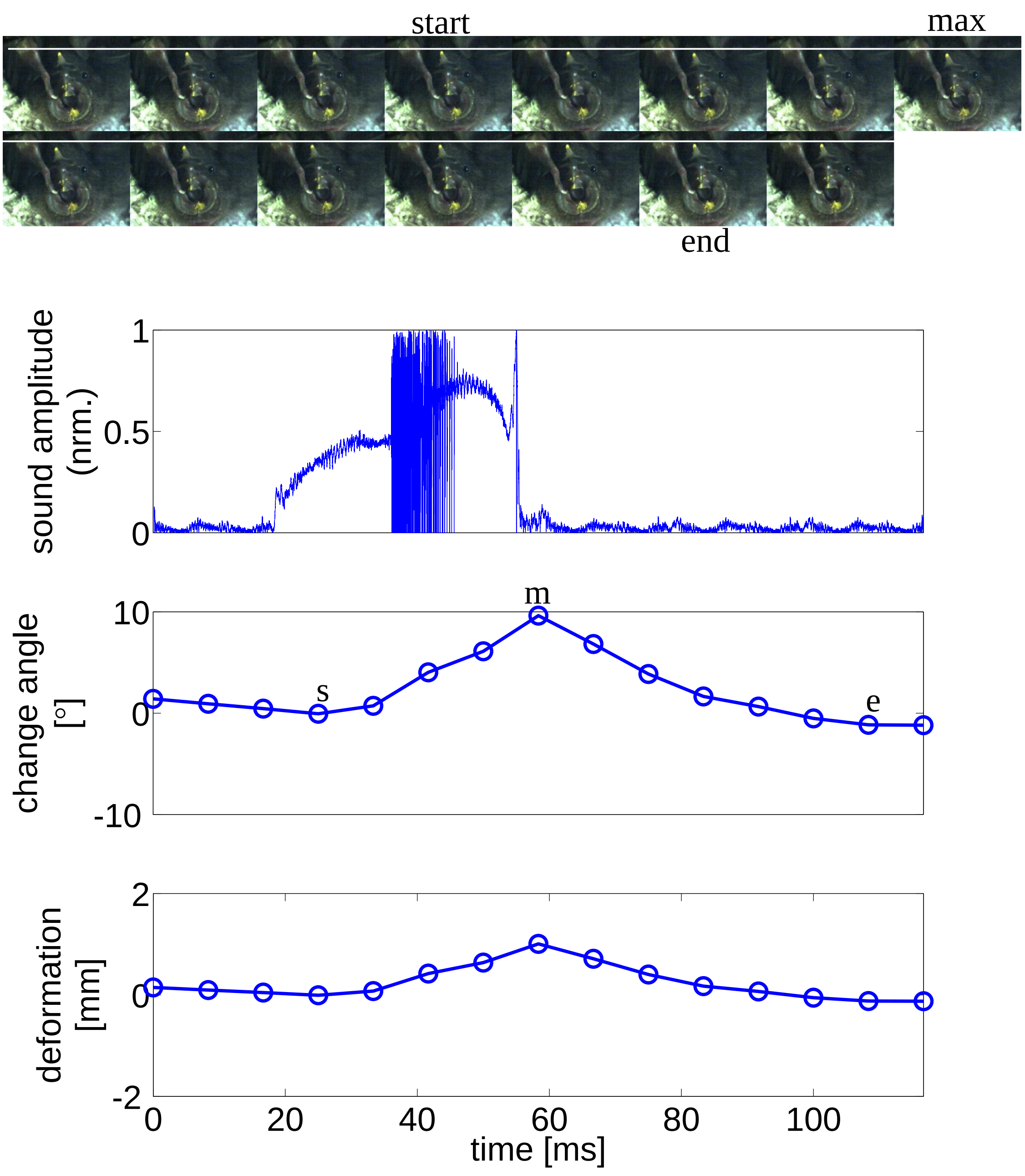

Recorded lancet motion sequence number 13.

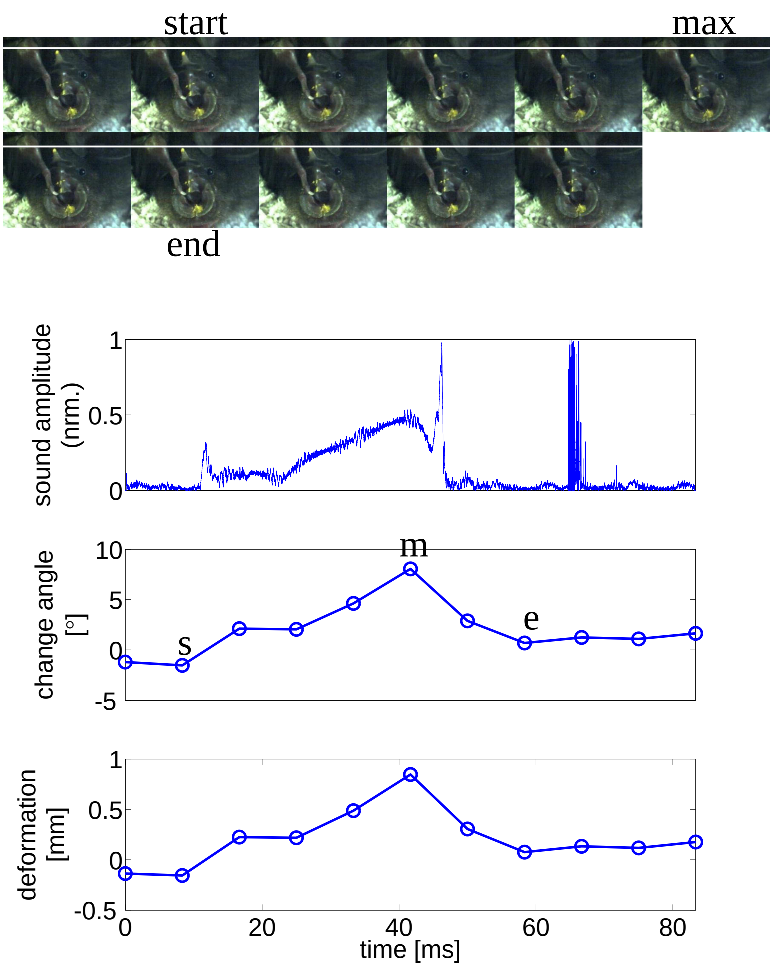

Recorded lancet motion sequence number 14.

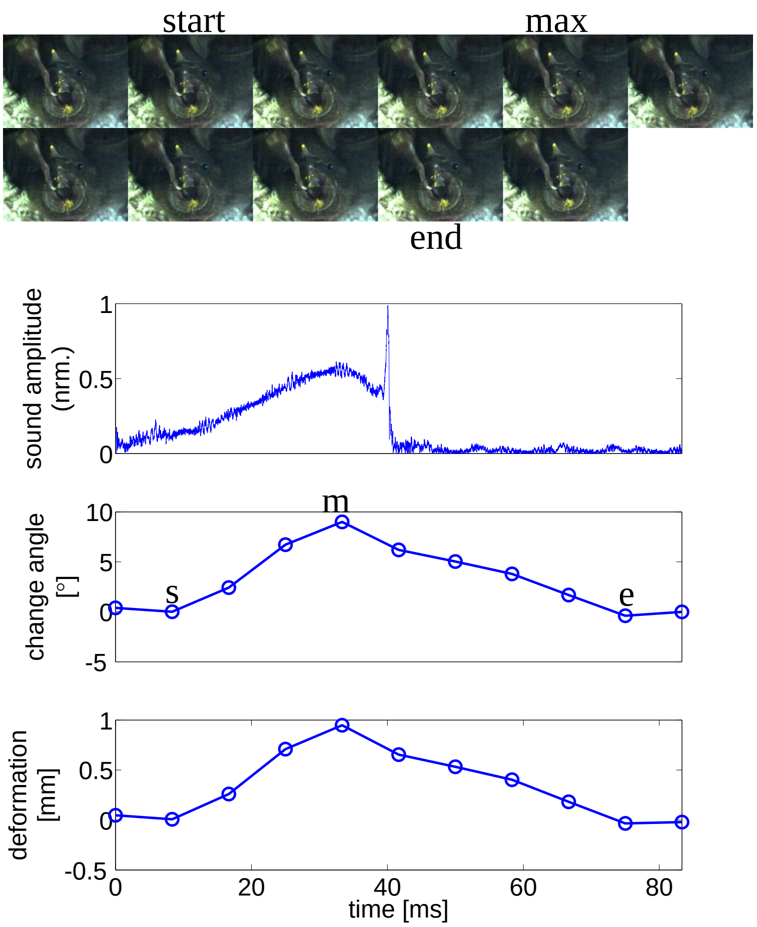

Recorded lancet motion sequence number 15.

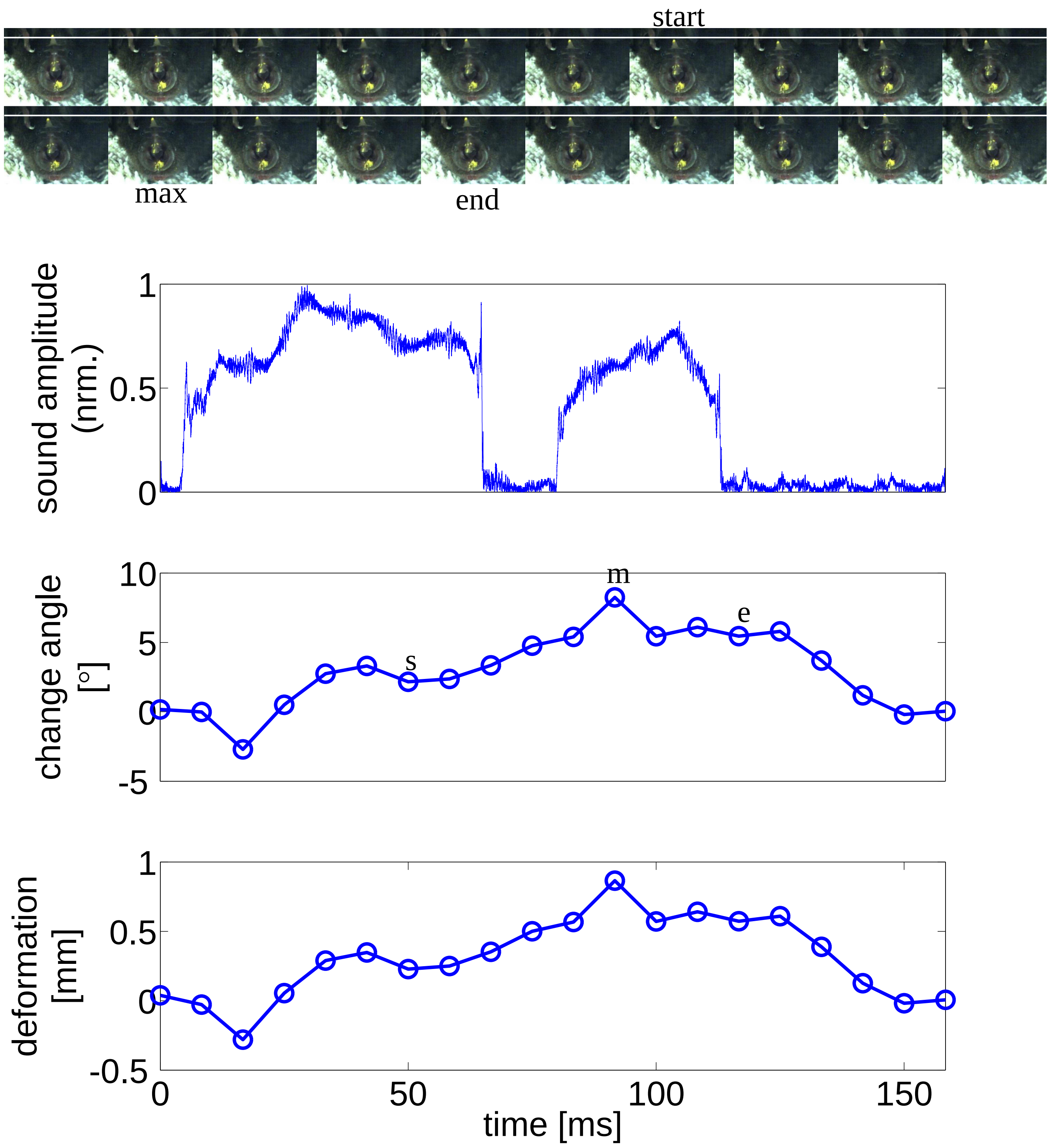

Recorded lancet motion sequence number 16.

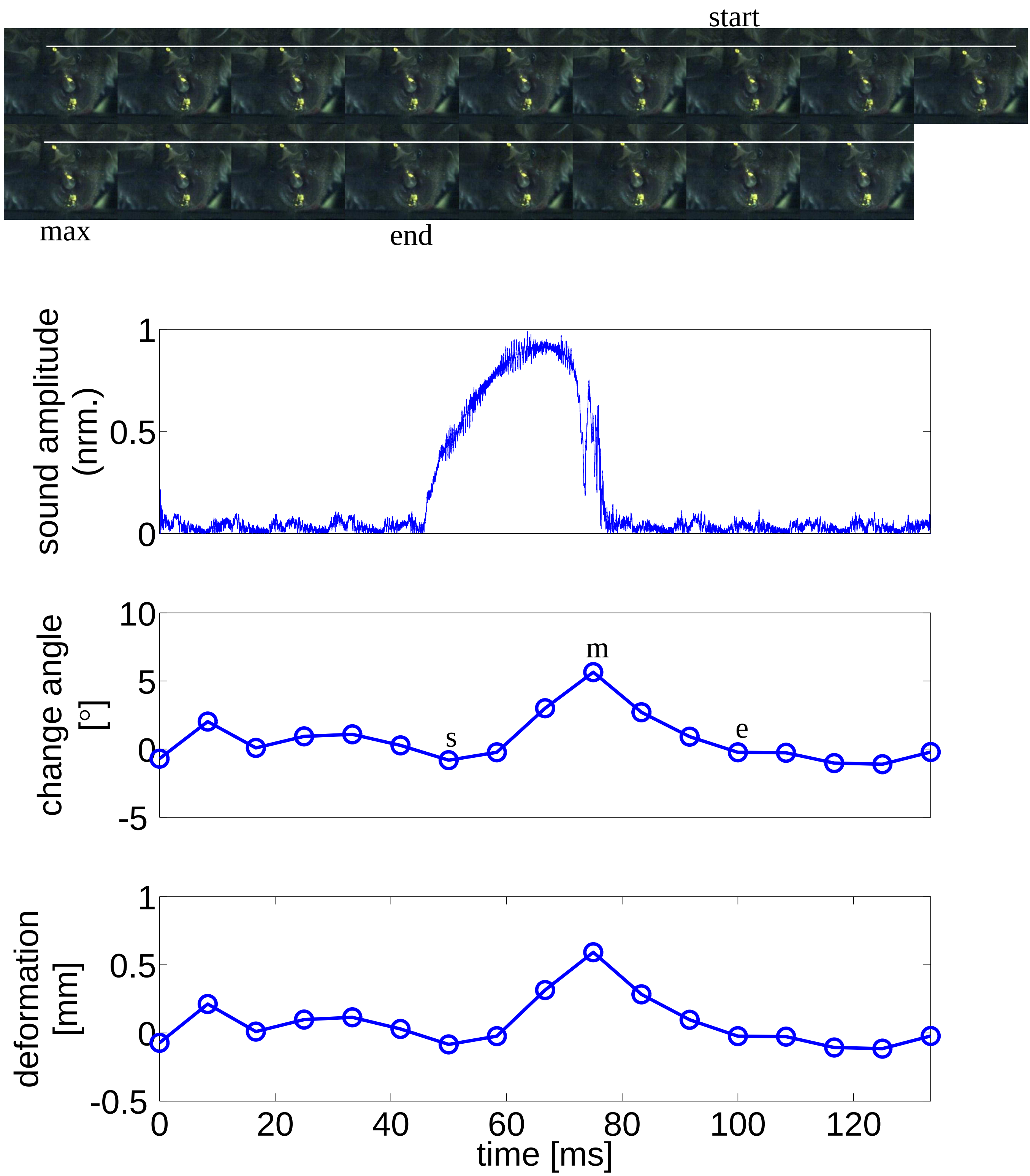

Recorded lancet motion sequence number 17.

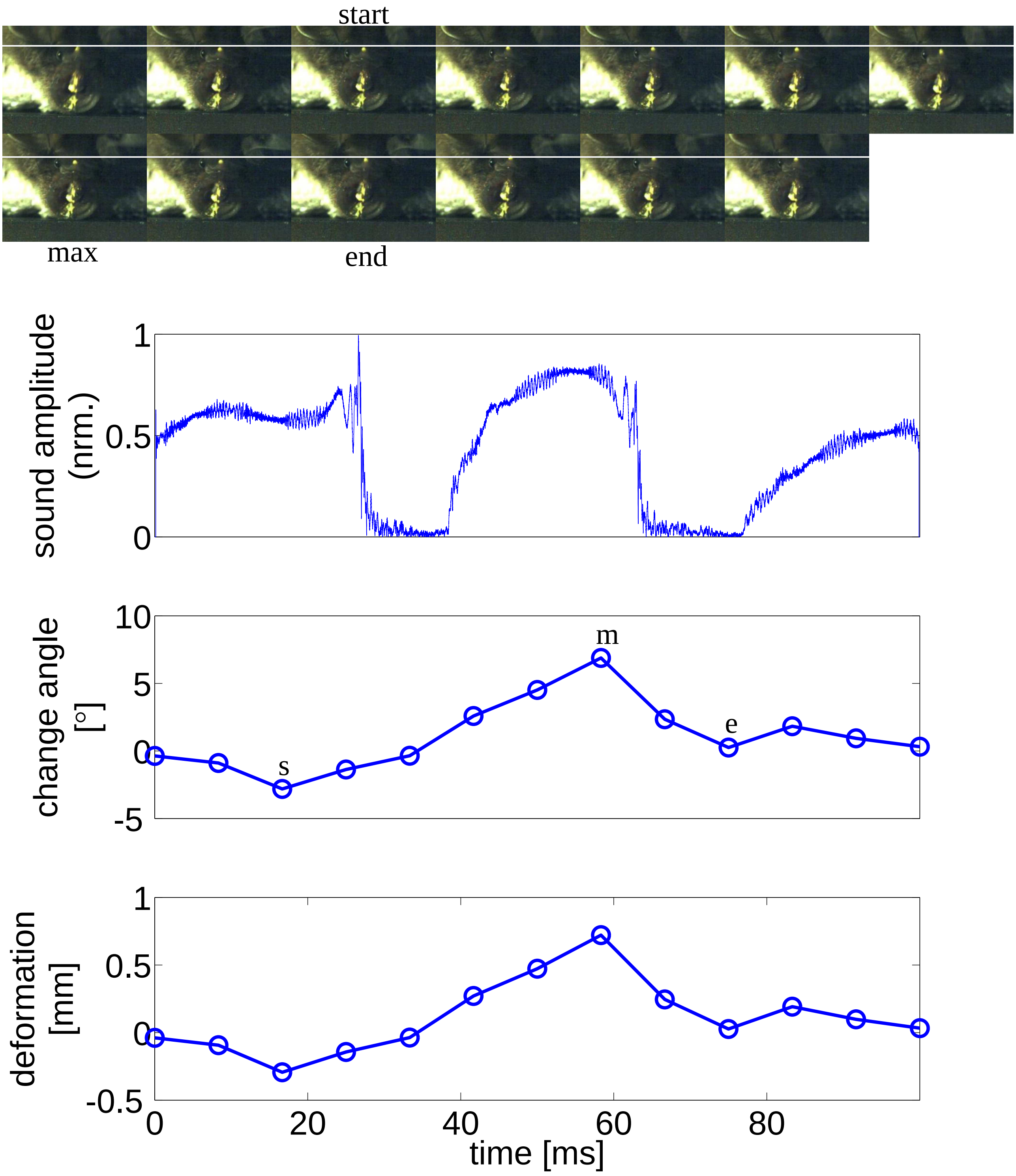

Recorded lancet motion sequence number 18.

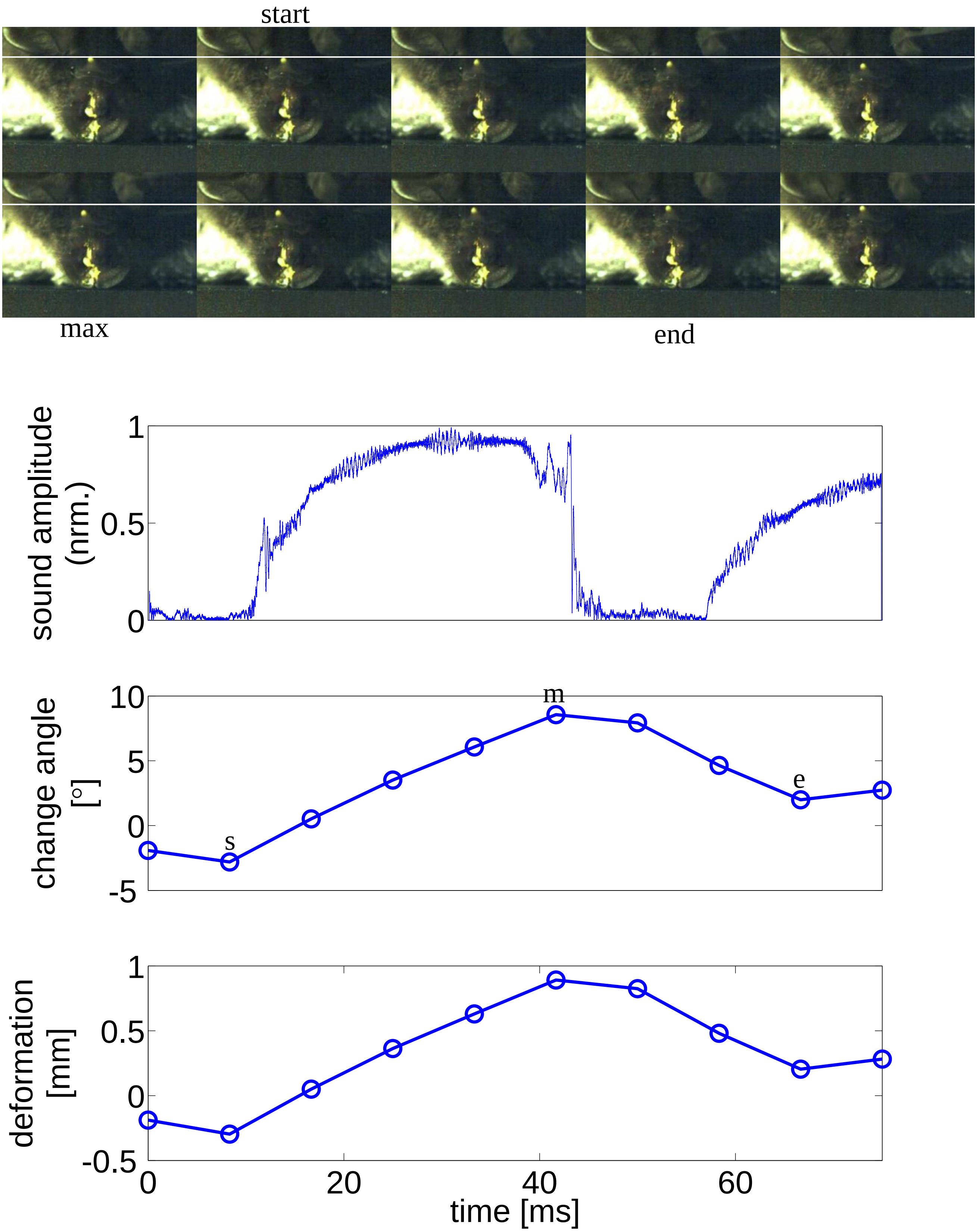

Recorded lancet motion sequence number 19.

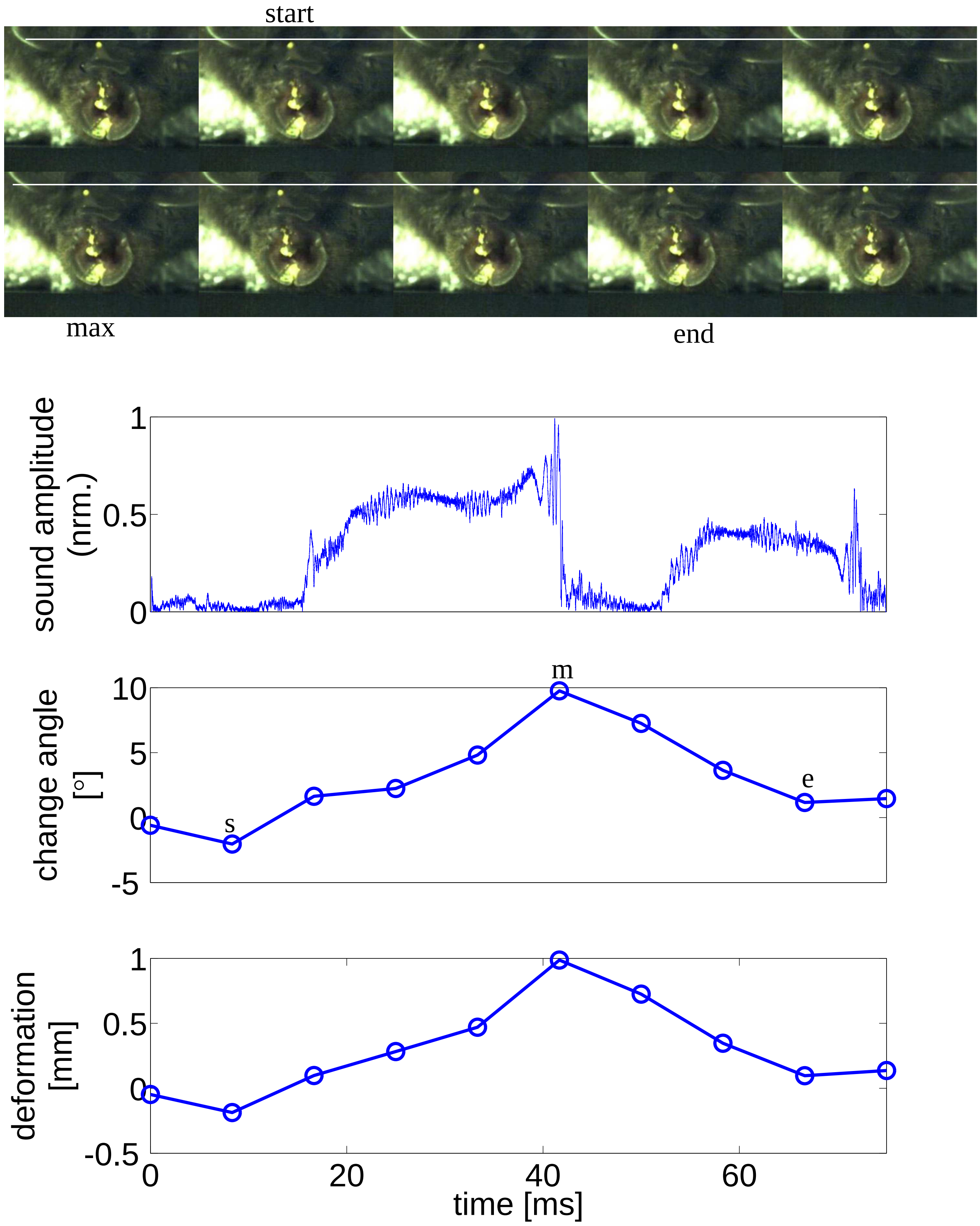

Recorded lancet motion sequence number 20.

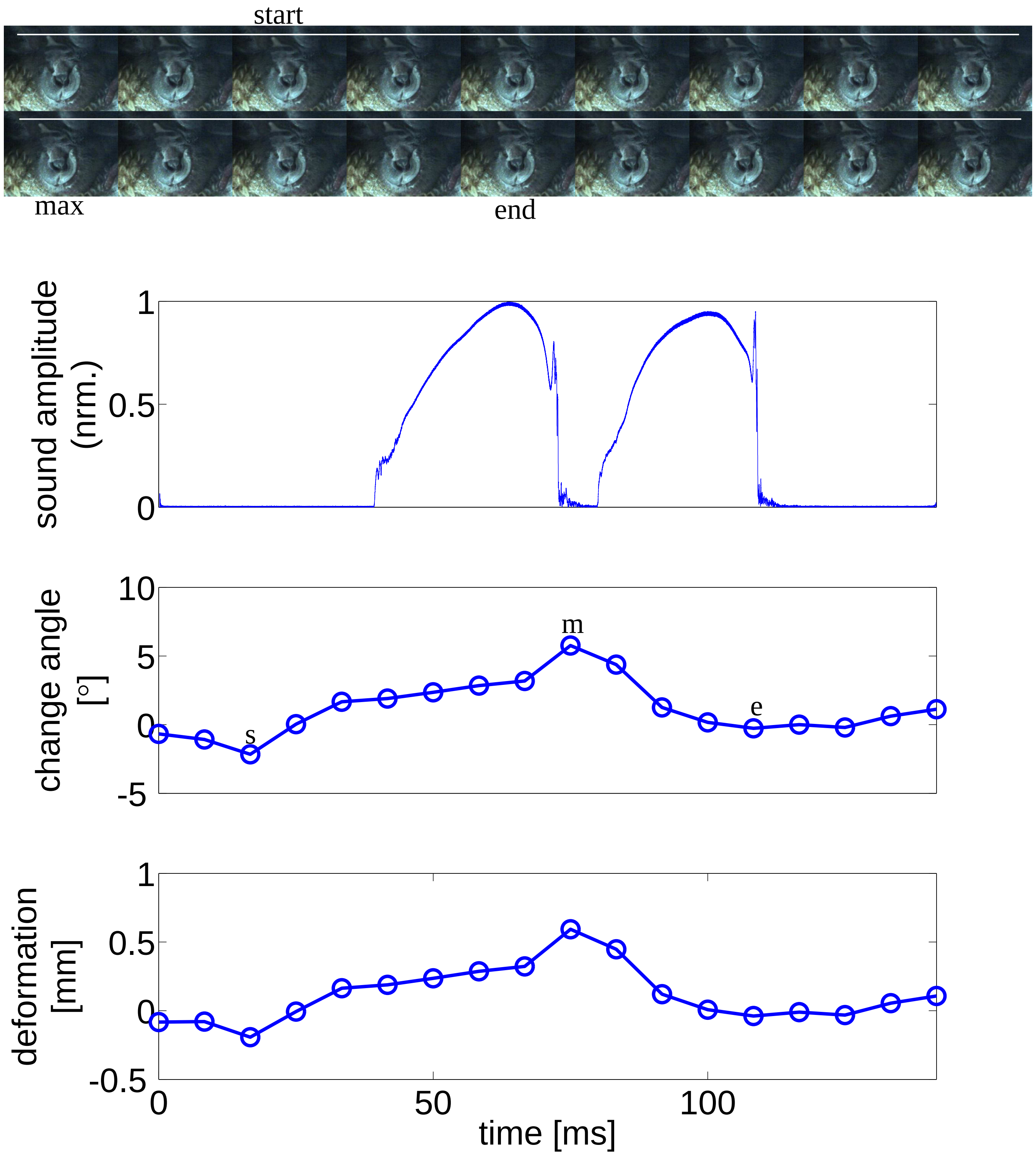

Recorded lancet motion sequence number 21.

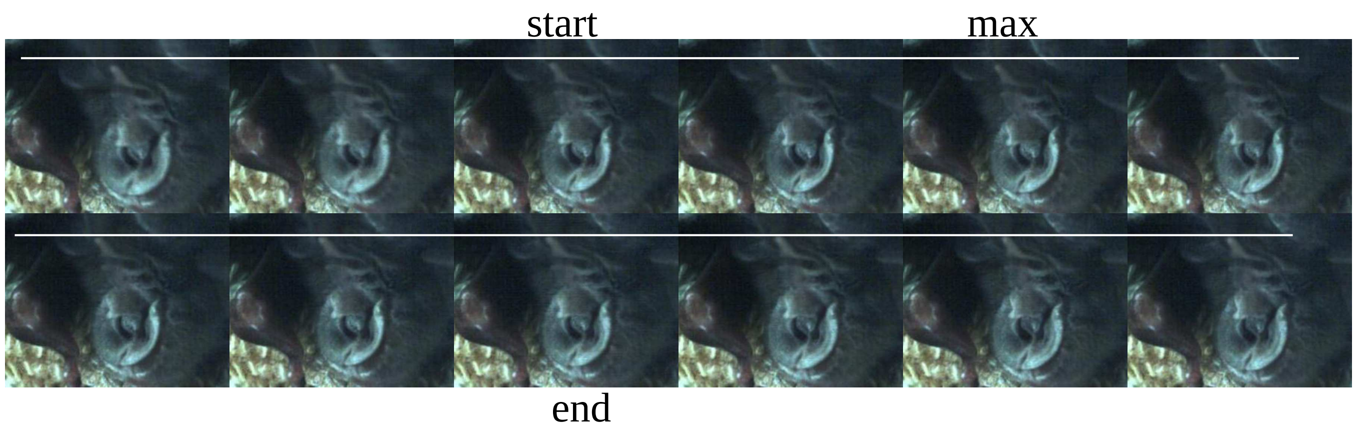

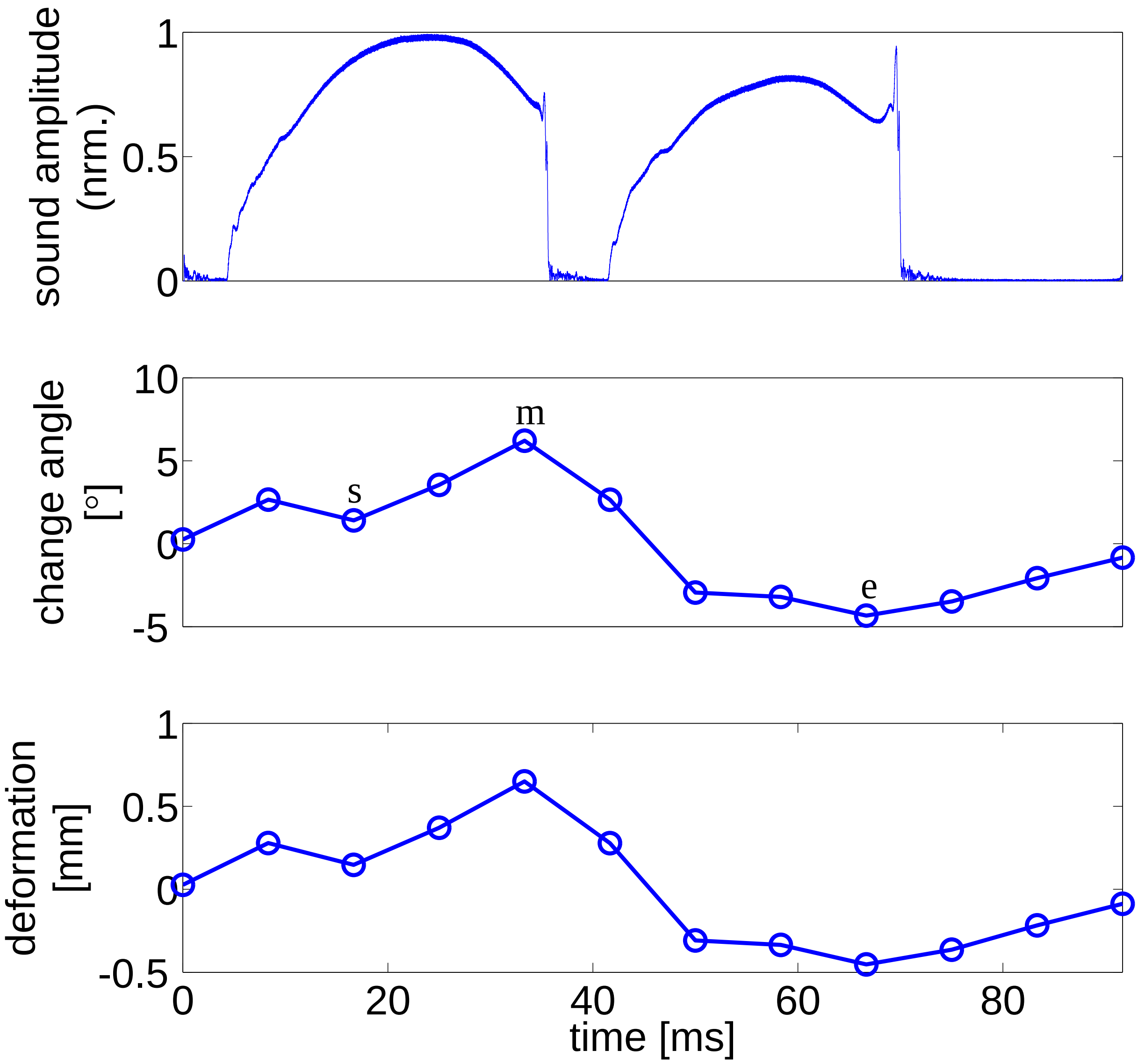

Recorded lancet motion sequence number 22.

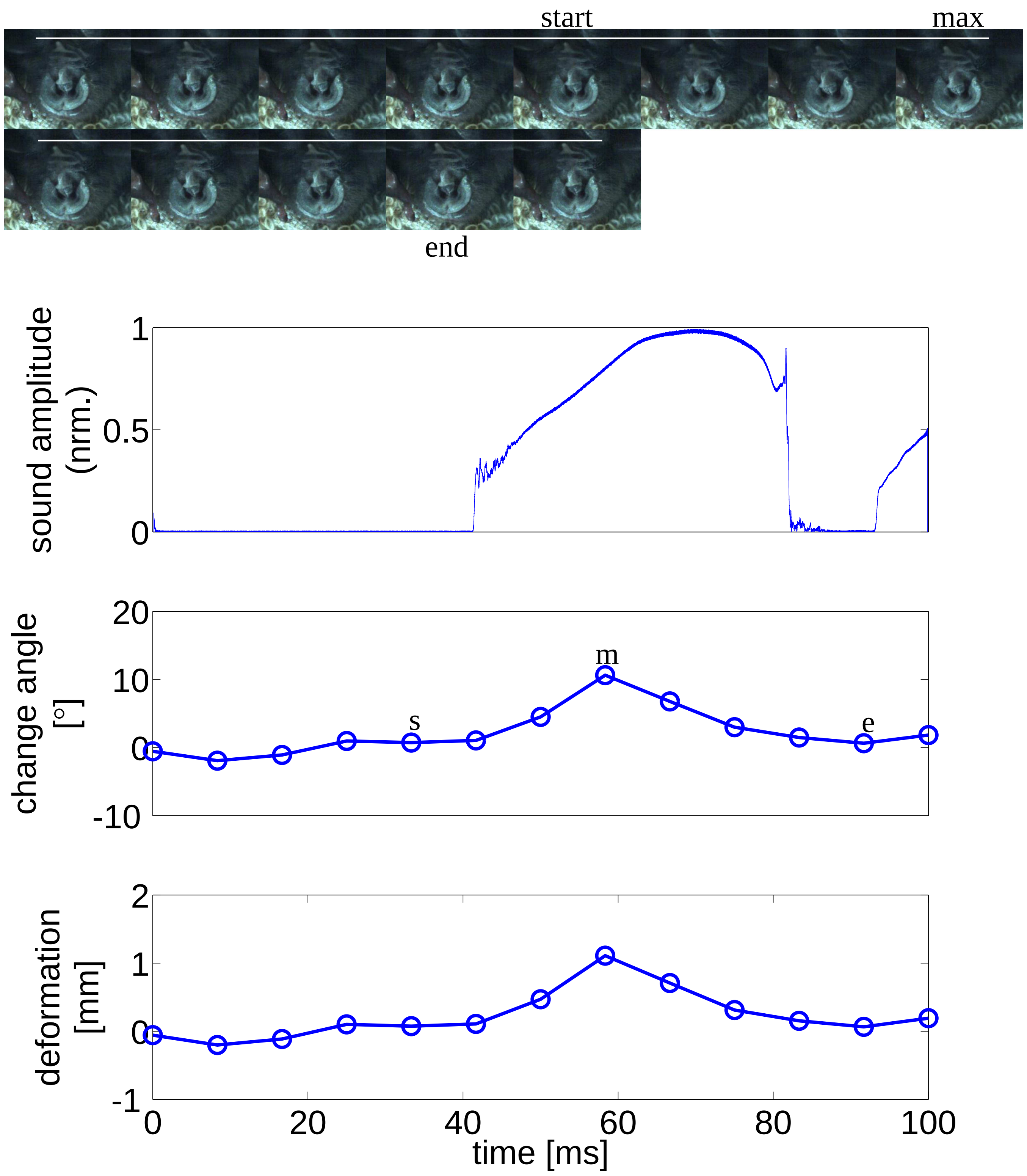

Recorded lancet motion sequence number 23.

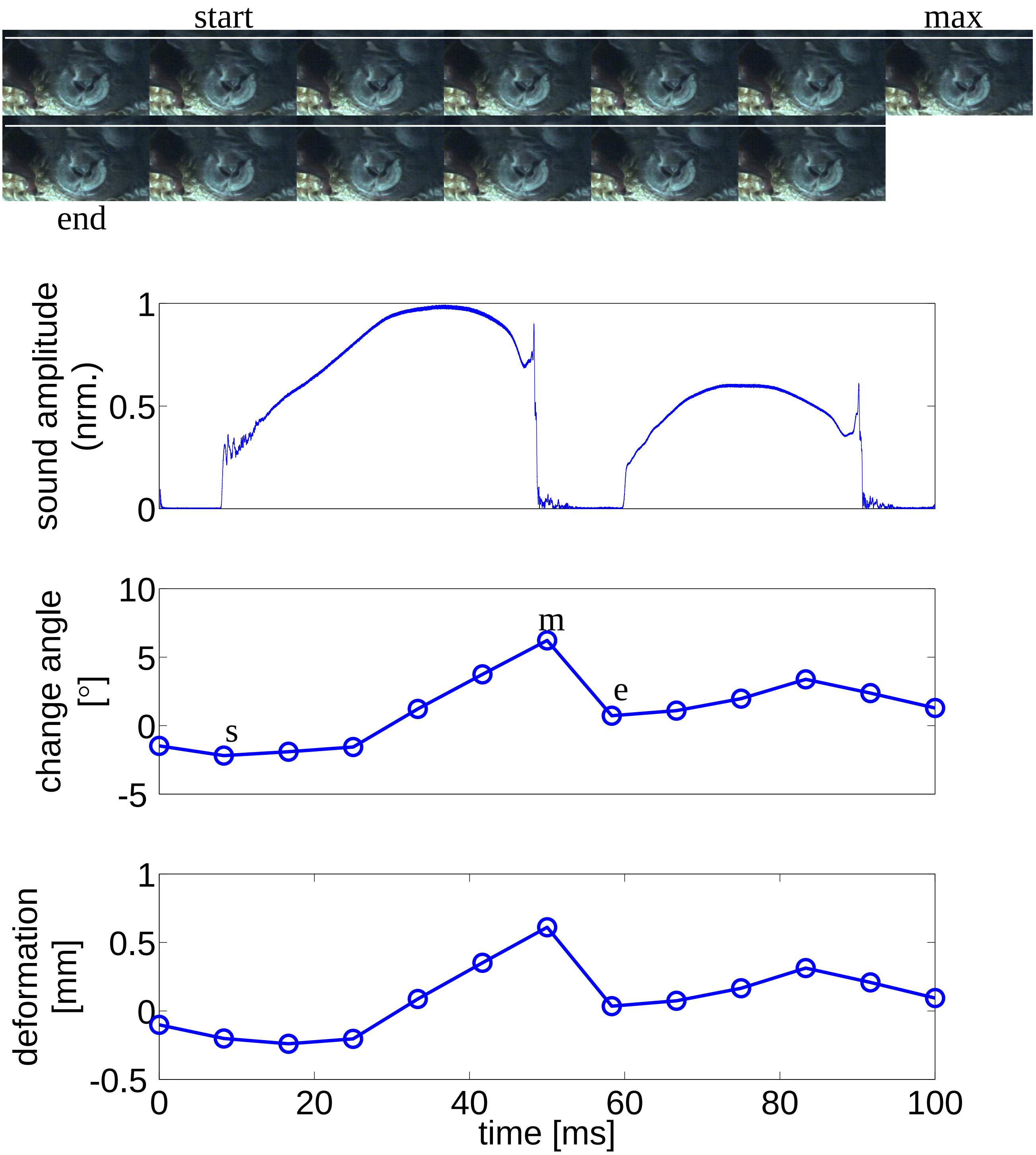

Recorded lancet motion sequence number 24.

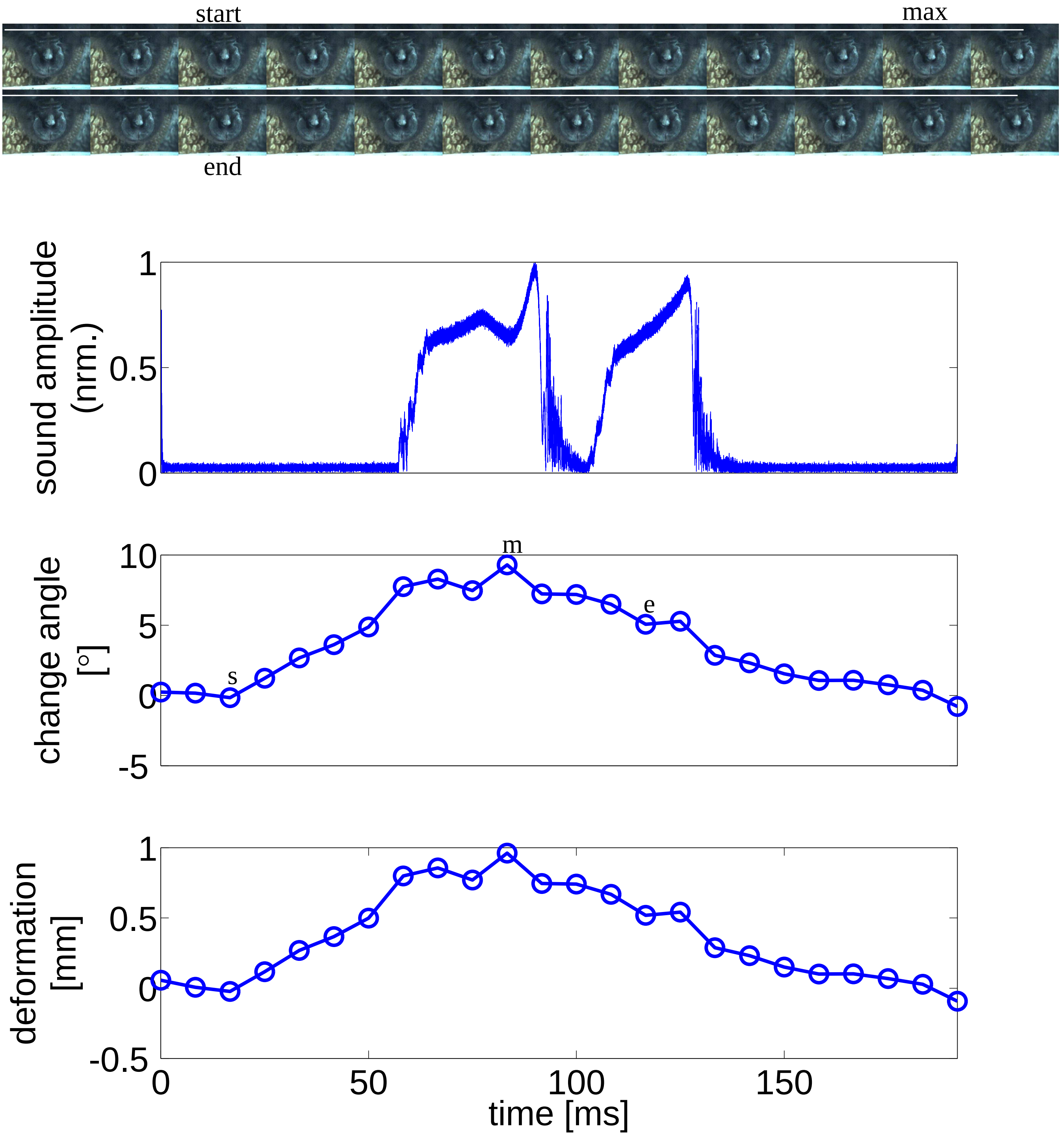

Recorded lancet motion sequence number 25.

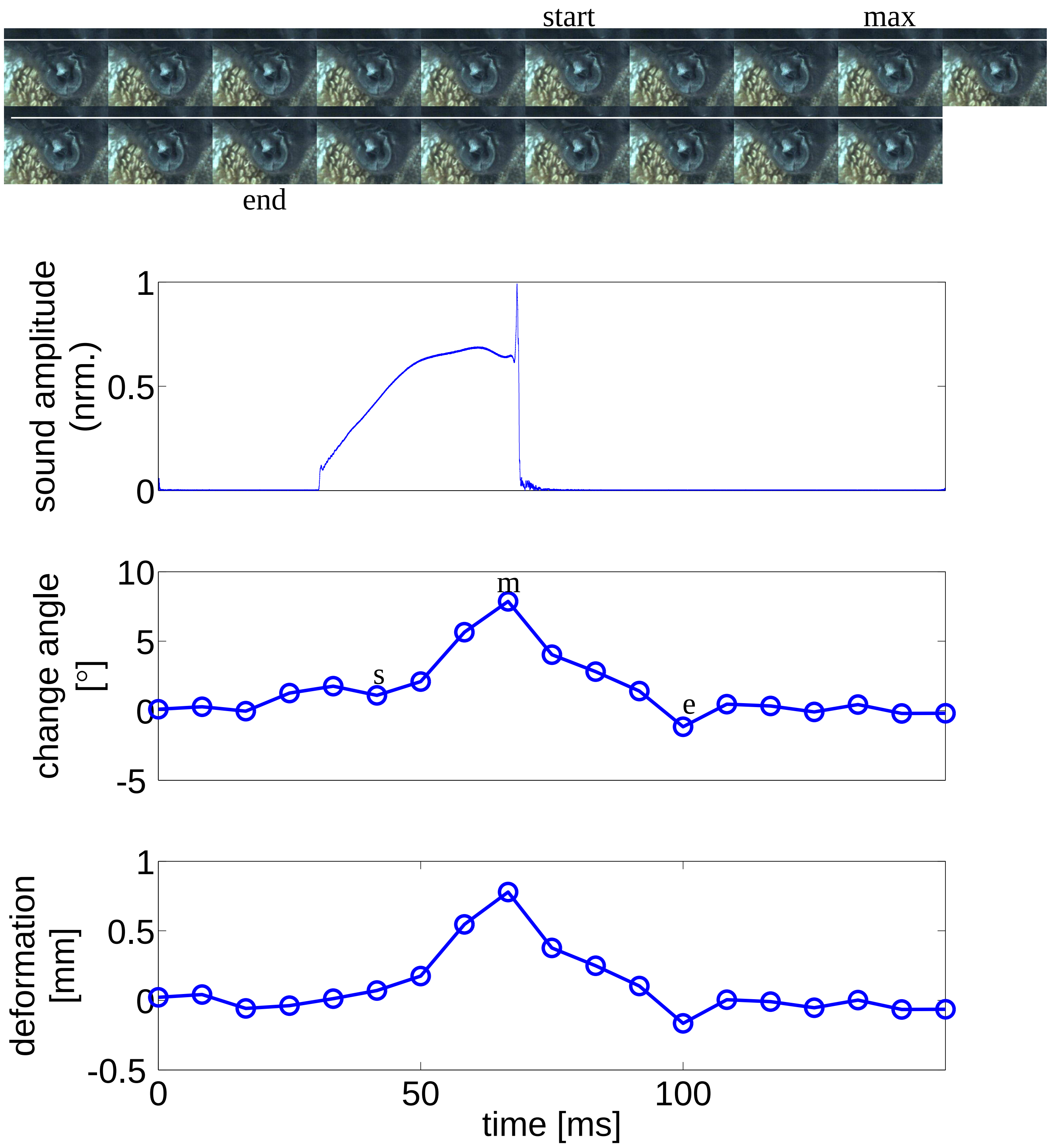

Recorded lancet motion sequence number 26.

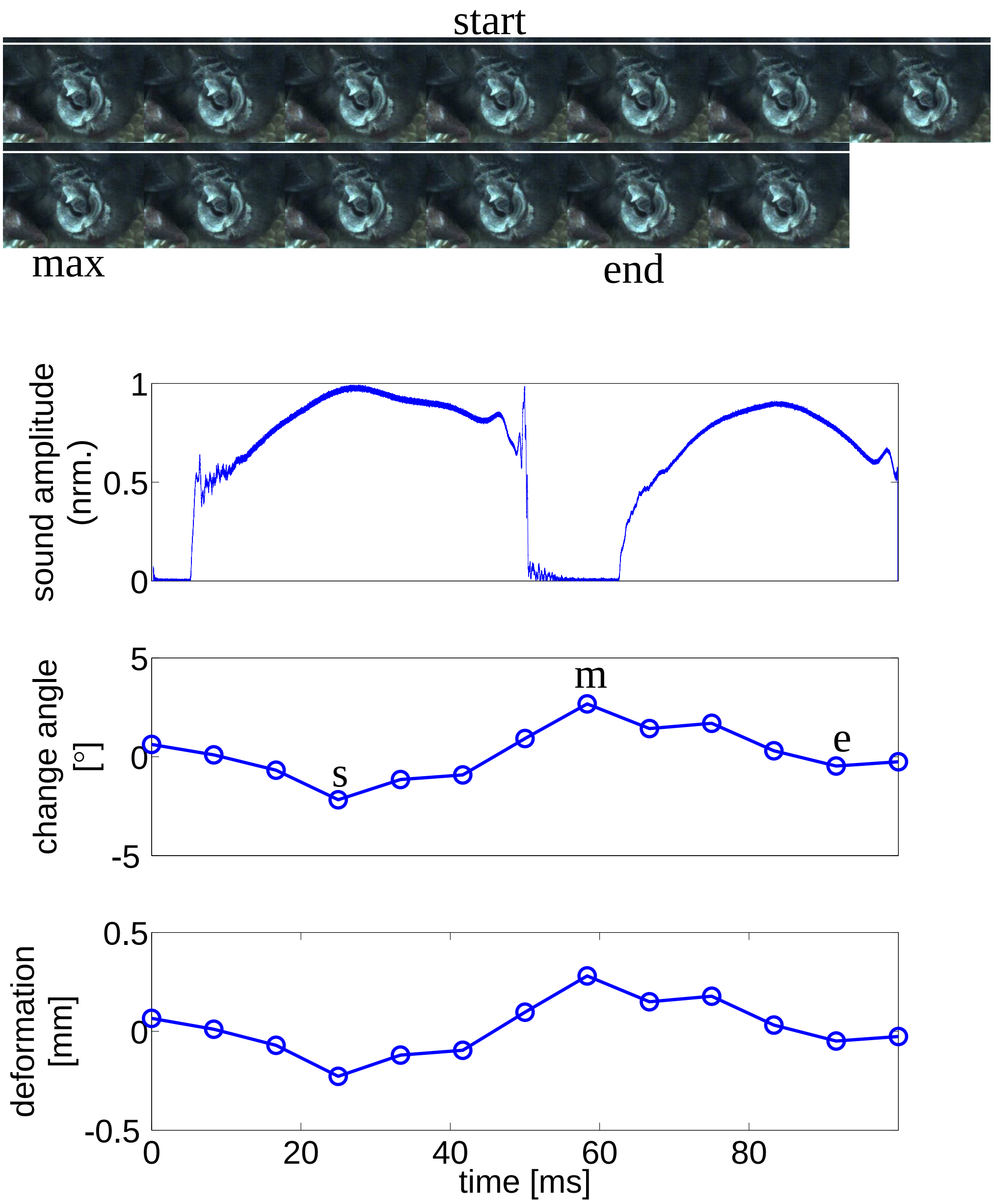

Recorded lancet motion sequence number 27.
